# Cancer-associated fibroblast-derived extracellular vesicles regulate lipophagy through PLIN2 to modulate dormancy in salivary gland adenoid cystic carcinoma cells

**DOI:** 10.1038/s12276-025-01600-3

**Published:** 2025-12-18

**Authors:** Zhichao Dou, Xu Zhang, Kun Meng, Mao Li, Xin Pang, Wanli Wang, Rongjia Shi, Xinhua Liang, Yaling Tang

**Affiliations:** 1 State Key Laboratory of Oral Diseases, National Clinical Research Center for Oral Diseases, Department of Oral Pathology, West China Hospital of Stomatology, Sichuan University, Sichuan, China; 2 State Key Laboratory of Oral Diseases, National Clinical Research Center for Oral Diseases, Department of Oral and Maxillofacial Surgery, West China Hospital of Stomatology, Sichuan University, Sichuan, China; 3 Department of Stomatology, The General Hospital of Western Theater Command PLA, Chengdu, China

**Keywords:** Cancer microenvironment, Metastasis, Head and neck cancer, Cell growth

## Abstract

Tumor recurrence and metastasis are largely attributed to dormant tumor cells receiving reactivation signals, particularly those originating from the tumor microenvironment. However, the detailed mechanisms of dormant tumor cell reactivation in salivary gland adenoid cystic carcinoma (SACC) remain largely unknown. Here our data revealed that autophagy is activated in dormant SACC cells but becomes downregulated once these cells are reactivated, and that cancer-associated fibroblast (CAF)-mediated autophagy promotes dormant SACC cells to resume proliferation and escape dormancy. Mechanistically, PLIN2 encapsulated in CAFs-derived extracellular vesicles promoted the initial stage of autophagy through the endoplasmic reticulum stress signaling pathway, and directly bound to p62 to promote lipid droplet degradation through the lipophagy pathway, which provided energy for the reactivation of dormant SACC cells. Moreover, we confirmed that PLIN2 expression was remarkably correlated with poor survival in patients with SACC. Finally, we verified that the combination of tozasertib and PLIN2 was stable through molecular docking and molecular dynamics simulation, indicating that tozasertib has the potential to serve as a targeted PLIN2 drug for CAFs in SACC. Our findings suggest that targeting PLIN2 and autophagy inhibition as part of primary SACC treatment may effectively eliminate dormant tumor cells and prevent SACC recurrence.

## Targeting PLIN2 to combat dormant salivary gland tumors

Salivary adenoid cystic carcinoma (SACC) is a type of cancer that often returns and spreads. Researchers wanted to understand how SACC cells survive in a dormant state and what causes them to become active again. Researchers used samples from patients and lab experiments to study SACC cells. They found that, when SACC cells are treated with chemotherapy, some enter a dormant state. These dormant cells use autophagy to survive. Cancer-associated fibroblasts, which are cells in the tumor environment, can send signals that reactivate these dormant cells. The study showed that cancer-associated fibroblasts release small particles called extracellular vesicles containing a protein called PLIN2. This protein helps dormant SACC cells use stored fats for energy, promoting their reactivation and growth. The findings suggest that targeting PLIN2 could help prevent SACC recurrence and improve treatment outcomes.

This summary was initially drafted using artificial intelligence, then revised and fact-checked by the author.

## Introduction

Salivary adenoid cystic carcinoma (SACC), a malignant tumor of the salivary glands, has unique biological characteristics, including extremely invasive and hematogenous metastasis^1,2^. Although surgery combined with adjuvant chemotherapy can improve the prognosis of patients with SACC, the recurrence rate remains 16–85%, and the rate of lung metastasis may reach 40%. Tumor recurrence and metastasis are the leading causes of patient mortality^1,3^. Chemotherapy and tumor surgery cannot completely kill tumor cells, and only a small number of residual tumor cells enter a dormant state. Tumor dormancy is an important stage in tumor development, during which dormant tumor cells retain the ability to re-enter a proliferative state. Their reactivation can ultimately lead to tumor recurrence and metastasis^4^. Therefore, exploring the molecular regulatory mechanism of SACC cell dormancy is of great value for improving clinical therapy.

Both intrinsic and extrinsic factors can affect SACC dormancy, which, if disturbed, may lead to apoptosis of dormant SACC cells, resumption of the proliferation program or escape from dormancy. Although tumor cells entering a dormant state significantly downregulate cellular metabolism, dormant tumor cells can maintain a metabolically active state by activating autophagy^5^. The crucial role of autophagy in cancer is complex, and the different functions of autophagy depend on various factors, including the stage of cancer and complex tumor microenvironment (TME)^6,7^. Autophagy activation is an intrinsic pathway that promotes the proliferation of dormant D2.0R (murine mammary neoplasia) cells, and autophagy inhibition can prevent the transition of dormant D2.0R cells from dormancy to growth^8^. However, the activation and effects of autophagy in dormant SACC cells have rarely been reported.

In addition to their specific intrinsic signaling pathways, dormant cancer cells acquire extracellular signals from the TME^9^. Cancer-associated fibroblasts (CAFs), the most abundant stromal cells in the TME, are closely associated with tumor progression. Numerous studies have confirmed that CAFs are key mediators of cancer dormancy and reactivation through paracrine regulation within the TME^10–12^. For example, a previous study reported that aging fibroblasts in the lung inhibit WNT5A signaling to induce the primary tumor by secreting sFRP1 to wake up dormant melanoma cells and promote the growth of metastatic tumors^13^. Moreover, the exosomes secreted by CAFs can transport a complete set of mitochondrial DNA (mtDNA) to dormant tumor cells, which in turn helps dormant tumor cells escape from dormancy and lead to tumor recurrence^14^. Nonetheless, the mechanisms underlying signaling communication between dormant SACC cells and CAFs have been rarely reported.

Abnormal lipid texture and continuous activation of lipid synthesis are important metabolic mechanisms in tumor cells^15^. A previous study found that, when energy and nutrients are scarce in the TME, CHKα2 in tumor cells can activate its protein kinase function and phosphorylate PLIN2/3. This modification allows the molecular chaperone HSC70 to recognize PLIN2/3, promoting the degradation of lipid droplets (LDs) and PLIN2/3 via the lipophagy pathway, thereby facilitating tumor initiation and progression^16^. Lipophagy is a selective autophagy that targets LDs and is an important regulatory mechanism for LD catabolism in addition to lipolysis through ATGL hydrolase^17^. Furthermore, PLIN2, an adipogenic differentiation-related protein, can indirectly participate in the synthesis of LDs^18^. In the TME, it remains unclear whether PLIN2 mediates crosstalk between CAFs and SACC cells via a paracrine mechanism to regulate SACC dormancy.

Our previous studies found that lipid metabolism reprogramming was necessary for hypoxia-induced SACC cell dormancy, while autophagy promoted tumor cell survival in the hypoxic TME^19,20^. In our study, we focused on the role of autophagy inhibition by pharmacological treatment or genetic intervention on the survival of dormant SACC cells. Specifically, we investigated the role of PLIN2 secreted by CAFs-derived extracellular vesicles (CAFs-EVs) in the reactivation of dormant SACC cells through the regulation of lipophagy in the TME.

## Materials and methods

### Patients and definition of clinical terms

Paraffin-embedded primary and recurrent SACC samples in 6 patients after postoperative chemotherapy, 48 paraffin-embedded SACC samples and 10 normal salivary gland (NSG) samples from 2012 to 2018 were collected from the Pathology Department of West China Stomatology Hospital, and the clinical pathological data of these 48 patients with SACC were compiled. In addition, ten pairs of frozen SACC tissues and matching paracancerous tissues archived by pathology at the West China Stomatology Hospital were obtained. The Ethics Committee of West China Hospital of Stomatology approved this study (WCHSRIB-D-2023-236).

### Cells and reagents

Normal fibroblasts (NFs) and CAFs were isolated from SACC and NSG tissues, respectively. SACC tissues were obtained from patients diagnosed with SACC, while NSG tissues were collected from patients undergoing surgery for sialolithiasis at West China Stomatology Hospital. This research was conducted in accordance with the Declaration of Helsinki and was approved by the Ethics Committee of West China Stomatology Hospital. Informed consent was obtained from all patients. Fresh tissue samples were washed with phosphate-buffered saline (PBS; Gibco) and chopped into small pieces (<1 mm^3^). The tissue pieces were then transferred to a culture flask and maintained in Dulbecco’s modified Eagle medium (DMEM; Gibco) with 10% fetal bovine serum (Gibco) and 100 units/ml penicillin–streptomycin (Gibco). Fibroblasts that migrated out of the tissue were isolated using trypsin. Fewer than ten generations of fibroblasts were used in the experiments. SACC-LM and SACC-83 cells were from the National Key Laboratory of Oral Disease Prevention and Control, West China Hospital of Stomatology. The cell medium was DMEM (Gibco) complete medium containing 10% FBS (Gibco) and 100 units/ml penicillin–streptomycin (Gibco) in an incubator with 5% CO_2_ at 37 °C.

Negative control and si-*ATG5* were produced by GenePharma. The sequence of si-*ATG5* was 5′-CGACGACGTTCGGTTCCGCCGTC-3′. Dormant SACC cells were transfected with pEZ-Lv201-*PLIN2* wild-type plasmid and pEZ-Lv201-PLIN2 plasmid using EndoFectin Max (GeneCopoeia). The *PLIN2* coding sequence was cloned into the pEV-Lv5 vector (GenePharma). Following the manufacturer’s instructions, SACC-LM cells with stable *PLIN2* overexpression were generated. GSK2606414, hydroxychloroquine (HCQ), tozasertib and cisplatin were purchased from MCE.

### Western blot and antibodies

Western blotting was performed as previously described^19^. In brief, proteins extracted from cells were separated by SDS–PAGE, transferred onto a polyvinylidene fluoride (PVDF) membrane, incubated with specific antibodies, and finally visualized using the Bio-Rad detection system. The following antibodies were used: anti-NR2F1 (ab181137) and anti-LC3 (ab192890) from Abcam; anti-Atg5 (12994), anti-p-mTOR (5536) and anti-p62 (16177) from Cell Signaling Technology; anti-caspase-3 (68773-1-Ig), anti-p-ATK (66444-1-Ig), anti-BAX (50599-2-Ig), anti-FAP (11779-1-AP), anti-BCL2 (12789-1-AP) and anti-α-SMA (14395-1-AP) from Proteintech; anti-HSP70 (ET1601-11), anti-GAPDH (ET1601-4), anti-CD63 (ET1607-2), anti-p21 (HA722685), anti-PLIN2 (ET1704-17), anti-p27 (ET1608-61) and anti-EIF2S1 (HA500385) from HUABIO; anti-p-PERK (340846), anti-PERK (R25311) from Zen-Bio.

### qRT–PCR

Total RNA was isolated from treated cells using TRIzol reagent (Takara), and cDNA was produced using a qRT–PCR kit (Takara) by reverse transcription. qPCR was performed using SYBR Green II (Bimake) and custom-designed primers (Sangon Biotech) on a QuantStudio 3 system (Thermo Fisher Scientific). The primer sequences used are presented in Supplementary Table 1.

### Immunofluorescence

For cell immunofluorescence (IF), cells were inoculated on a glass slide and given different treatments. Cells were fixed and permeabilized, followed by blocking in 2% bovine serum albumin. Then, cells were incubated with appropriate antibodies. Slides were dyed with fluorochrome-conjugated second antibody the next day. Nuclei were stained with 4′,6′-diamidino-2-phenylindole (DAPI) and imaged using an Olympus FV3000 inverted confocal microscope (Olympus).

For IF of tumor sections, after the paraffin sections were dewaxed to water, the antigen was repaired and the autofluorescence signal was quenched. Slides were incubated with 2% bovine serum albumin and the appropriate antibodies. The next day, the slides were incubated with fluorophore-conjugated secondary antibodies. Nuclei were stained with DAPI, and the samples were observed using an Olympus FV3000 confocal microscope (Olympus).

### CCK-8 assay and colony formation assay

A total of 1–4 × 10^3^ SACC cells were inoculated in a 96-well plate. Each experimental group was inoculated with six wells. After different treatments, Cell Counting Kit-8 (CCK-8) reagent (Biosharp) was added to the cells. Finally, the optical density was measured by enzymoleter at 450 nm, and the cell proliferation and survival rate were calculated.

For cell colony formation analysis, 500–1,000 dormant SACC cells transfected with si-*ATG5* or si-*NC* were seeded in a well (6-well plates). After about 1–2 weeks of culture, the colonies were fixed and stained using multifocus formaldehyde and crystal violet, respectively. The clone formation rate was calculated from the images captured by the microscope and camera.

### Apoptosis and cell cycle assay

After the different treatments, SACC cells were suspended in 1× binding buffer (200 μl) and stained with Annexin V-FITC and propidine iodide (PI) in the dark (eBioscience). Samples were examined using a flow cytometer (Thermo) and assessed using FlowJo software.

After the different treatments, SACC cells were collected for cell cycle analysis and fixed with 95% ethanol. After centrifugation, staining was performed with 500 μl propyl iodide (Dojindo Laboratories) in the dark. The cell samples were assessed by flow cytometry (Thermo) and calculated using FlowJo software.

### Three-dimensional cell culture

The experimental methods were performed as previously described^20^. Specifically, dormant SACC cells were cultured in DMEM (Gibco) medium containing 2% Matrigel (Corning) in 96 wells precoated with Matrigel. The cells were then treated with HCQ or CAFs- derived conditioned medium (CAFs-CM) and photographed using a Leica microscope.

### Calcein-AM/PI costaining

A calcein-AM/PI staining kit (Solarbio) was used for SACC cell staining. In brief, the cells were seeded on slides and incubated with different treatments. Then, cells were stained with calcein-AM/PI and observed using fluorescence microscopy (Leica).

### Immunohistochemistry

Immunohistochemistry (IHC) analysis and quantitation of results were performed as previously reported^21^. Briefly, after paraffin removal and antigen repair, the tissues were incubated with primary and secondary antibodies respectively, and stained using a DAB kit (Solarbio) and hematoxylin. Images were captured using an Olympus microscope. Tissue sections were scored as follows: percentage of positive cells: 0% = 0, 0–1% = 1, 2–10% = 2, 11–30% = 3, 31–70% = 4 and 71–100% = 5. Staining intensity was scored as follows: negative = 0, weak = 1, moderate = 2, strong = 3. The total score was calculated by combining the percentage and intensity scores.

### TUNEL assay

The mouse tumor sections were stained using a TUNEL assay kit (Solarbio) to observe cell apoptosis, following the manufacturer’s instructions. Images were obtained by fluorescence microscopy (Leica).

### Collection of conditioned medium

For the collection of conditioned medium (CM), 1 × 10^6^ CAFs or NFs were seeded overnight in a 100-mm Petri dish containing DMEM supplemented with 10% FBS. After 24 h, the culture medium was changed to DMEM with 0.5% FBS. After another 24 h, the CM was collected. The CM was purified with a 0.2-μm filter (Millipore) and preserved in a −80 °C refrigerator for subsequent experiments.

### RNA sequencing

To investigate the mechanism by with CAFs secretion factors regulate SACC dormancy, we performed whole-transcriptome sequencing on three CAFs and three NF samples from SACC or NSG tissues. Total RNA from NFs or CAFs was extracted using TRIzol reagent, followed by RNA sequencing analysis. The GENE DENOVO System (Guangzhou GENE DENOVO Biotechnology) was used to perform quality inspection of the RNA. After mRNA interruption, synthesis of the first strand of cDNA, cDNA end repair and screening, a library was contructed. The GENE DENOVO system was then used for library quality inspection. Subsequently, the cDNA library was sequenced using an Illumina HiSeqTM 2000 platform.

### Transmission electron microscopy (TEM) and nanoparticle tracking analysis (NTA)

For observation of autophagosomes, cells were inoculated into 6-well plates at 1.0–2.5 × 10^5^ cells per well and transfected with PLIN2 overexpression plasmid or CAFs-CM. Cells were fixed with 2.5% glutaraldehyde at pH 7.4, then fixed with 1% osmium tetroxide. After staining with 1% uranyl acetate, the cells were dehydrated and embedded in epoxy resin. The samples were polymerized in an oven, and ultrathin slices were obtained using a Leica microtome. The sections were stained and observed under a transmission electron microscope (JEOL) at an accelerated voltage of 80 kV. Photographs were captured and analyzed by Chengdu Lilai Biotechnology.

To observe extracellular vesicles, the extracellular vesicles were suspended in 4% paraformaldehyde (PFA) and observed using TEM (JEM-1230, JEOL). The size and number of extracellular vesicles were investigated using a NanoSight NS300 system (NanoSight).

### Wound healing and invasion assays

When the cells reached confluence in six-well plates, a wound was created using a 200-μl pipette tip. The medium was then replaced with NFs-derived conditioned medium (NFs-CM) or CAFs-CM, and images of the wound were captured at 0 h and 24 h. Cell migration was analyzed using ImageJ software.

For the invasion assay, the dormant cells were suspended in 100 μl serum-free medium and inoculated in the upper Matrigel-coated transwell chamber. NFs-CM or CAFs-CM was added into the lower chamber. The cells were fixed with 4% PFA and stained with 0.1% crystal violet, counted and photographed under the microscope.

### Extracellular vesicle isolation and purification

After NFs or CAFs reached 90% confluence, they were cultured in DMEM containing extracellular vesicle-depleted FBS for 48 h. CM was collected and filtered using a 0.22-μm filter (Millipore). CM was centrifuged at 300*g* for 10 min, 2,000*g* for 10 min and 10,000*g* for 30 min and filtered through a 0.22-μm filter (Millipore). Extracellular vesicles were then enriched by ultracentrifugation (Beckman) at 100,000*g* for 2 h at 4 °C and stored at −80 °C for subsequent experiments.

### Extracellular vesicle labeling and uptake assay

Extracellular vesicles were stained with red fluorescent dye 1,1'-dioctadecyl-3,3,3',3'-tetramethylindocarbocyanine perchlorate (Dil) (Beyotime) and washed twice to remove the free dye. Then, Dil-labeled extracellular vesicles (200 μg/ml) and dormant SACC cells were seeded in DMEM with 10% FBS (excluding extracellular vesicles) for 6 h and 24 h, respectively. Dormant SACC cells were stained with green fluorescent phalloidin (Abcam) to visualize the cytoskeleton, and nuclei were counterstained with DAPI After staining, the samples were observed under an Olympus confocal fv3000 microscope (Olympus).

### Bioinformatics analysis

To analyze candidate gene expression in SACC tissues, we obtained the datasets GSE153002 and GSE88804 from the Gene Expression Omnibus (GEO) database (https://www.ncbi.nlm.nih.gov/geo/). Combined with previously reported literature^22^ (the population studied in this literature included 54 patients with SACC), the differentially expressed genes were analyzed for clinical prognosis, and Kaplan–Meier survival curves were plotted using GraphPad Prism.

### Nile Red staining

To visualize the accumulation of intracellular LDs, cells were fixed with 4% PFA and stained with Nile Red (Beyotime) at 37 °C, and nuclei were counterstained with DAPI. Images were captured using Olympus inverted confocal fv3000 (Olympus, Japan).

### Co-immunoprecipitation

Co-immunoprecipitation (co-IP) was performed to identify interaction between p62 and PLIN2 on SACC cells. In brief, cells were lysed at 4 °C in a cell IP buffer containing 150 mM NaCl, 1% Nonidet P-40, 0.1% sodium deoxycholate, 100 mm NaF, 5 mM MgCl_2_, 0.5 mM Na_3_VO_4_, 0.02% NaN_3_, 0.1 mM 4-(2-aminoethyl)-benzenesulfonyl fluoride and a 1.0% protease inhibitor cocktail. As recommended by the manufacturer, the total protein was immunoprecipitated with 2.0 μg p62 specific antibody plus protein agarose beads (Beyotime) overnight. The immunoprecipitated proteins were separated by SDS–PAGE and transferred onto a polyvinylidene fluoride membrane, which was then blocked in PBST containing 5% dry milk for 1 h. The nonprecipitated supernatant was used as input. Membranes were then incubated with antibodies against PLN2 and p62. The washed bands were incubated with horseradish peroxidase-conjugated anti-IgG. The bands were observed with an ECL chemiluminescence detection kit (MCE).

### Protein structure modeling, molecular docking and molecular dynamics simulation

Given the incomplete crystal structure of PLIN2, we generated its theoretical structure using the homology modeling tool SWISS-MODEL (https://swissmodel.expasy.org/). Furthermore, we retrieved the three-dimensional structure of P62 (PDB ID: 5YP7) from the Research Collaboratory for Structural Bioinformatics Protein Data Bank (RCSB PDB; https://www.rcsb.org/) database. The protein structure was established by AutoDockTools software to ensure the precision of the molecular docking. Protein–protein docking was performed using GRAMM, and the resulting protein–protein complexes were optimized using AutoDockTools software. PLIN2–p62 interactions were predicted by PyMOL software.

Molecular dynamics simulations were performed using Desmond/Maestro software (version 2022.1). TIP3P water molecules were added to the complex system using the TIP3P water model, and then a 0.15 M NaCl solution was used to balance the system. The treatment systems were allowed to interact electrostatically and minimize their energy, followed by a 100-ns molecular dynamics simulation under isothermal–isobaric equilibrium conditions. The control simulation temperature was 300 K, and the pressure was 1 bar.

### In vivo experiments

The animal study was approved and performed in compliance with the guidance suggestion of Animal Care Committee of West China Hospital, Sichuan University (permit number 220230523002). The BALB/c nude mice (5 weeks old) used in this experiment were purchased from Beijing Huafukang Biotechnology. The animals were raised in the Animal Center of West China Hospital for 1 week before conducting experiments. First, GFP-SACC-LM cells were induced into dormancy by cisplatin treatment in vitro, and then dormant GFP-SACC-LM and NFs or CAFs were co-injected into the flank skin at a ratio of 5:1. Each nude mouse was injected with approximately 1 × 10^7^ cells. One week after inoculation of cells, tumor formation under the skin of mice could be observed. The tumor growth, tumor size and the weight of mice were observed and recorded.

For the construction of the nude mouse lung metastasis model, first, GFP-SACC-LM was induced into dormancy by in vitro cisplatin treatment, the cell suspension was prepared by trypsin digestion and the concentration was adjusted to 1 × 10^6^/200 μl. The injection volume used was 1 × 10^6^. Three days after tail vein injection in nude mice, group treatment was performed: the control group received NFs-EVs via tail vein, while the experimental group was administered CAFs-EVs. EVs were administered at a dose of 10 μg every 3 days. The body weight of the nude mice was monitored and recorded, and a body weight change curve was plotted. Lung metastasis in nude mice was detected using small-animal in vivo imaging. Five weeks after the tail vein injection of dormant GFP-SACC-LM cells, the mice were euthanized, and gross specimens of the lung, liver and kidney were collected for analysis.

The SACC-LM cell line stably transfected with PLIN2 was injected subcutaneously into nude mice. Approximately 1 × 10^7^ cells were injected into the mice. When the primary tumor reached about 500 mm^3^ after 4 weeks, the primary tumors of eight mice were resected and randomly divided into two groups. One group was given 2 weeks of cisplatin treatment (3 mg/kg) twice weekly. One group was given 2 weeks of combined treatment with cisplatin and HCQ (cisplatin at 3 mg/kg, HCQ at 20 mg/kg) twice a week. Both cisplatin and HCQ were administered via intraperitoneal injection. The tumor size and recurrence were observed and measured. After 2 weeks of administration, the administration was no longer continued. The tumor growth curve of nude mice was drawn.

GFP-labeled NC-SACC-LM and PLIN2-SACC-LM cells (1 × 10^6^) were injected into mice through the tail vein. After 3 days, all mice were treated with cisplatin for 2 weeks. The body weights of the mice were recorded, and BLT AniView100 multi-mode animal in vivo imaging system was used for in vivo imaging. After 5 weeks, the mice were euthanized and the lung tissues were collected for H&E staining to observe metastases.

### Statistical analyses

All in vitro experiments were repeated at least three times. Statistical analysis was performed in GraphPad Prism 9.0 software using unpaired *t*-tests, paired *t*-tests or one-way analyses of variance where appropriate. The correlations between the PLIN2 staining score and clinicopathological factors were examined using Spearman correlation analysis. The Kaplan–Meier method was used to evaluate the survival curves of patients with SACC. Statistical significance was determined using the log-rank test. ^*^P < 0.05, ^**^P < 0.01, ^***^P < 0.001; ns, *P* ≥ 0.05.

## Results

### SACC cells entered a dormant state upon activating autophagy

**Fig. 1:**
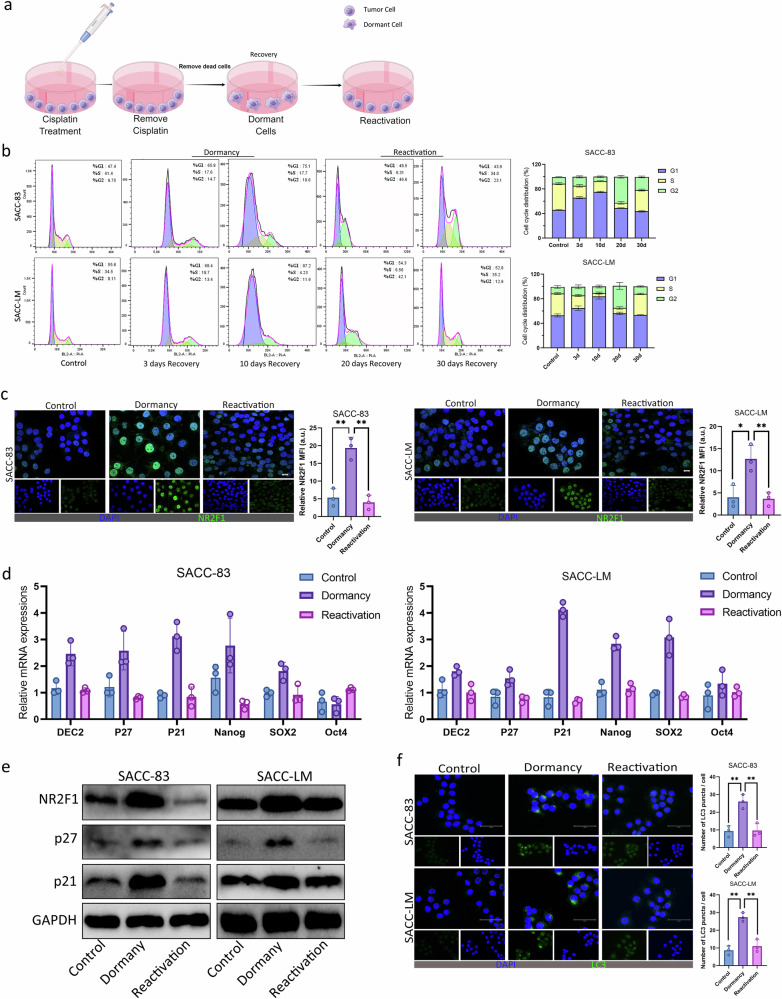
SACC cells entered a dormant state upon activating autophagy. **a** Schematic diagram of cisplatin-induced dormancy. **b** Cell cycle analysis of dormant and reactivated SACC cells. **c** IF analysis for the NR2F1 in the dormant SACC cells and reactivated SACC cells. Scale bar, 50 μm. **d**, **e** qRT–PCR (**d**) and western blot (**e**) for the indicated genes in the dormant SACC cells and reactivated SACC cells. **f** IF analysis for the LC3 in the dormant SACC cells and reactivated SACC cells. Scale bar, 50 μm.

In clinical practice, postoperative adjuvant chemotherapy often fails to completely eliminate tumor cells; the residual cells can enter a dormant state, ultimately leading to tumor recurrence and metastasis^23^. We established an model of dormant SACC cells in vitro through short-term cisplatin treatment based on previously published protocols^24–26^ (Fig. 1a). After cisplatin (2 μM) treatment for 48 h, most SACC cells were killed and the remaining surviving cells showed a mesenchymal-like shape. After 20 days of cisplatin removal, SACC cells returned to their original state (Supplementary Fig. 1a). Most cells that survived chemotherapy were arrested in the G0–G1 phase with high expression of dormancy maker NR2F1, while approximately 20 days after removing cisplatin, SACC cells entered the S phase and G2/M phase with low expression of NR2F1 and began to regrow (Fig. 1b and Supplementary Fig. 1b), suggesting that cisplatin treatment could induce SACC cells to enter dormancy. Similarly, 3,3′-dioctadecyloxacarbocyanine perchlorate (Dio) staining confirmed that SACC cells entered a cellular dormant state after 48 h of cisplatin treatment (Supplementary Fig. 1c). Furthermore, the expression levels of cell dormancy markers DEC2, NR2F1, P27, P21, Nanog and SOX2 increased in dormant SACC cells, whereas the expression levels of dormancy markers in reactivated SACC cells recovered correspondingly (Fig. 1c–e). Tumor samples were obtained from patients with SACC who experienced recurrence after surgery and chemotherapy, and the reactivated status of the tumor cells was confirmed clinically by Ki-67 staining (Supplementary Fig. 1f). The above results show that cisplatin treatment (2 μM) caused SACC cells to enter a dormant state, and dormant SACC cells could be reactivated after removing cisplatin to achieve reversal of the dormant phenotype.

Studies have reported that autophagy is involved in tumor dormancy, and we assessed the levels of LC3/MAP1LC3 in dormant SACC cells. Consistent with autophagy activation, dormant SACC cells showed a significant increase in LC3 fluorescent spots and LC3 levels. Interestingly, dormant tumor cells were no longer in an autophagy activated state after reactivation (Fig. 1f and Supplementary Fig. 1d). Furthermore, cisplatin treatment inhibited the phosphorylation of AKT protein and mTOR, indicating that the inhibition of the AKT pathway caused the high expression of P27, which in turn led to SACC cell cycle arrest and an increase in NR2F1 abundance. Moreover, the mTOR pathway inhibition increased the conversion of LC-I to LC3-II, thereby activating autophagy in dormant SACC cells (Supplementary Fig. 1e). Therefore, we concluded that autophagy in dormant SACC cells was activated when SACC cells entered a dormant state.

### Activation of autophagy was a means to maintain the survival of dormant SACC cells

**Fig. 2:**
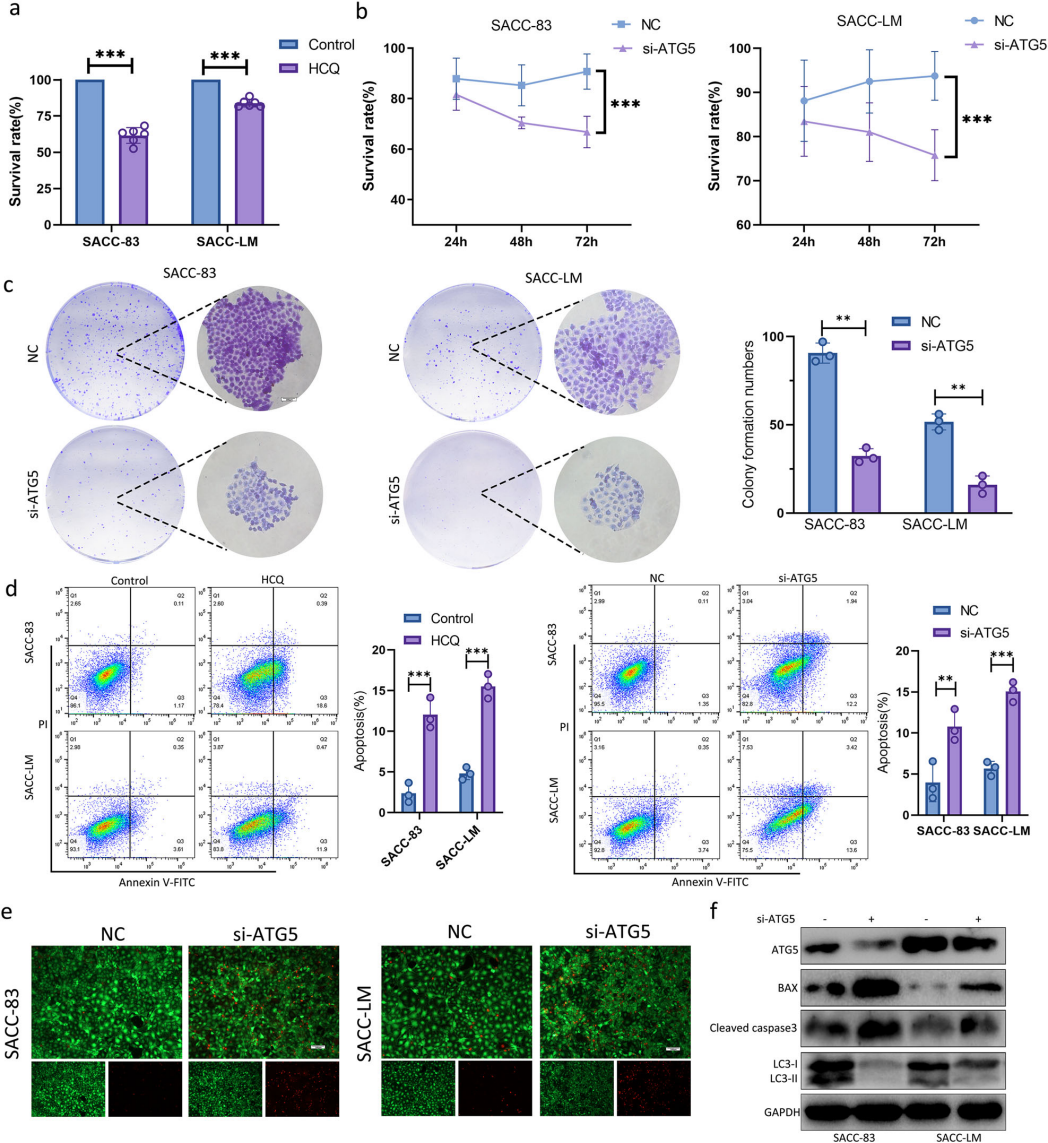
Activation of autophagy was a means to maintain the survival of dormant SACC cells. **a**,**b** The survival analysis of dormant SACC cells treated with HCQ (20 μM) (**a**) or transfected with si-ATG5 (**b**). **c** Cell clonal formation assay of dormant SACC cells transfected with si-ATG5. Scale bar, 100 μm. **d** Apoptosis analysis of dormant SACC cells treated with HCQ or transfected with si-ATG5 using Annexin V-FITC/PI staining. **e** Cell survival analysis of dormant SACC cells transfected si-ATG5 by calcein-AM/PI costaining. Scale bar, 100 μm. **f** Western blot for the indicated proteins in the dormant SACC cell transfected with si-ATG5.

To explore the function of autophagy in dormant SACC cells, drug intervention with HCQ (an autophagy inhibitor) for 24 h was used to inhibit autophagic flux of dormant SACC cells. We found that the proportion of surviving dormant SACC cells decreased significantly (Fig. 2a). We also used gene interference to knockdown the key autophagy gene *ATG5* in dormant SACC cells to inhibit the autophagy process. Similarly, the proportion of surviving dormant SACC cells also decreased significantly (Fig. 2b). To visualize the apoptosis and proliferation of dormant SACC cells under these conditions, we performed cell cloning experiments and cell flow cytometry to detect apoptosis. After ATG5 expression was inhibited, the colony-forming ability of dormant SACC cells was significantly reduced. Moreover, both HCQ treatment and *ATG5* knockdown significantly increased the apoptosis rate of these cells (Fig. 2c,d). Similar results were obtained from live/dead staining, where si-ATG5 inhibited the survival of dormant SACC cells. (Fig. 2e). Western blot analysis showed that, after silencing *ATG5* in dormant SACC cells, the expression of BAX and cleaved caspase-3 increased (Fig. 2f). To better simulate the TME, we also conducted a three-dimensional spheroidization assay and found that HCQ treatment inhibited the spheroidization ability of dormant SACC cells (Supplementary Fig. 2a–c). The results suggested that autophagy promoted the survival of dormant SACC cells.

### CAFs may promote the reactivation of dormant SACC cells upregulating autophagy

**Fig. 3:**
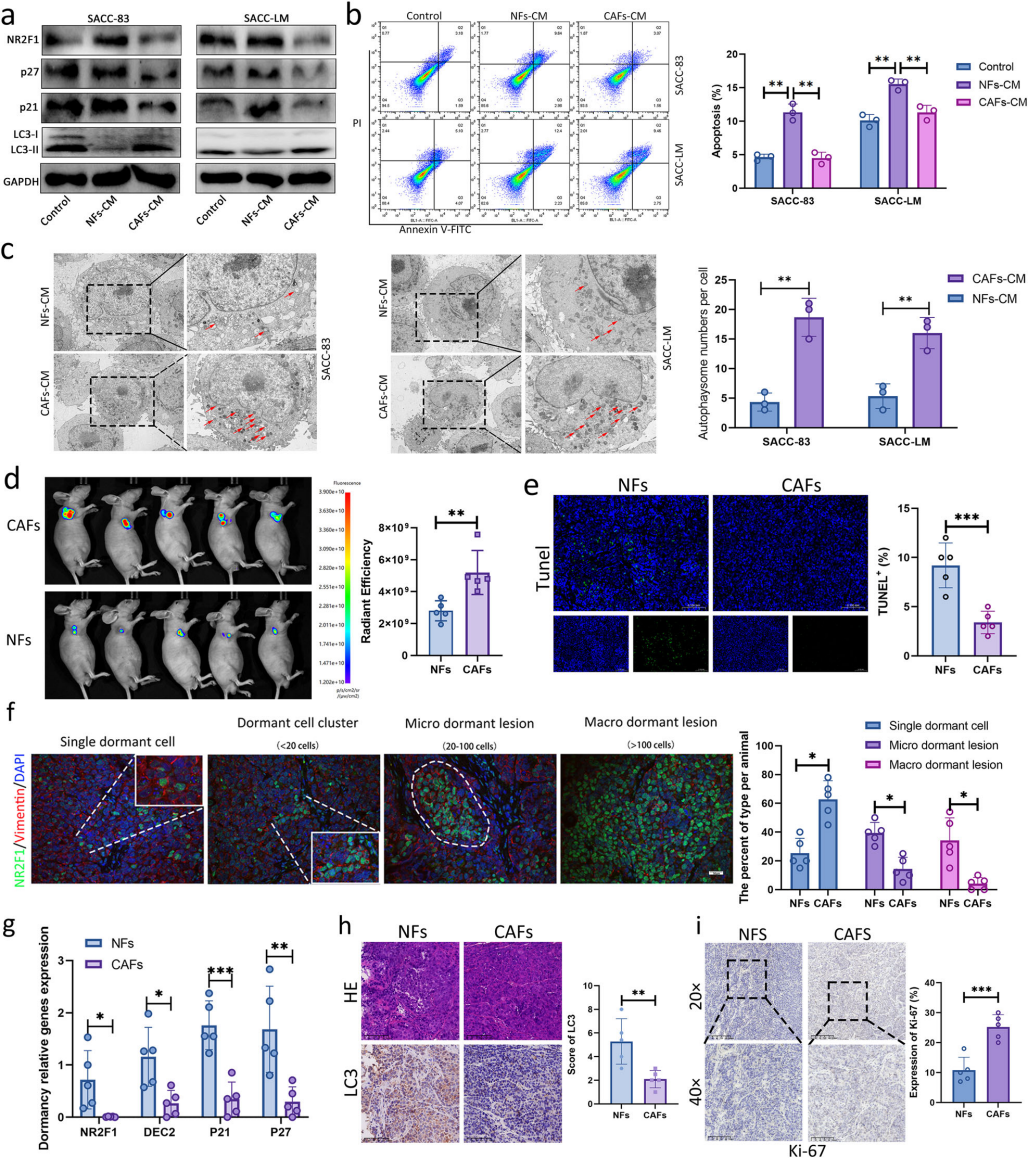
CAFs may promote the reactivation of dormant SACC cells upregulating autophagy. **a** Western blot for the indicated proteins in the dormant SACC cell treated with NFs-CM or CAFs-CM. **b**,**c** Apoptosis analysis (**b**) and TEM (**c**) of dormant SACC cells treated with NFs-CM or CAFs-CM. **d** Dormant GFP-SACC-LM cells with CAFs or NFs were intrahepatically injected into athymic nude mice (*n* = 5 per group). The primary tumors of nude mice were imaged. **e** TUNEL assay of tumor tissues in nude mice. Scale bars, 100 μm. **f** IF representative images show the stain for NR2F1 (green), vimentin (red) and DAPI (blue) in primary tumor tissues. Scale bars, 50 μm. **g** qRT–PCR measured the relative mRNA level of dormancy in primary tumor tissues. **h**,**i** IHC analysis of xenograft tumors from mice was performed to examine the expression of LC3 (**h**) and Ki-67 (**i**). Representative staining images are shown.

Increasing evidence indicates that CAFs do not exist merely as isolated cells around tumors^27^, but actively communicate with tumor cells to promote malignant behaviors. Therefore, we first hypothesized that CAFs in the tumor may be involved in regulating the reactivation of dormant SACC cells. To test this hypothesis, we successfully extracted and identified NFs and CAFs with high FAP and α-SMA expression (Supplementary Fig. 3a–d). The Ki-67-positive rate of dormant SACC cells treated with CAFs-CM was significantly increased (Supplementary Fig. 4a), the migration and invasion abilities were also enhanced (Supplementary Fig. 4b,c) and CAFs-CM treatment reduced the level of NR2F1, p27 and p21, indicating that CAFs-CM had a certain reactivation effect on dormant SACC cells (Fig. 3a). Moreover, the CAFs-CM played a positive role in protecting the survival of dormant SACC cells and resisting apoptosis (Fig. 3b). Interestingly, CAFs-CM increased the number of autophagosomes and LC3 level in dormant SACC cells (Fig. 3a–c). This finding was inconsistent with the above results, which suggested that autophagy serves to maintain dormancy. To explain this phenomenon, we conducted in vivo experiments.

Then, we conducted subcutaneous xenograft tumor experiments in mice to test whether CAFs can affect the fate of dormant SACC cells in vivo. First, SACC-LM cells were treated with cisplatin for 48 h in vitro to establish dormant SACC-LM cells and then injected subcutaneously into mice with NFs or CAFs at a ratio of 5:1. Tumor tissues were collected 24 days later. The tumor weight and growth rate of the CAF group were higher than those of the NF group (Fig. 3d and Supplementary Fig. 4d,e). Furthermore, TUNEL staining showed that coculture with CAFs significantly reduced the proportion of apoptosis in dormant SACC cells (Fig. 3e). We analyzed the proportion of dormant cells by labeling dormant cells with the NR2F1 antibody. Compared with NFs, CAFs resulted in a higher frequency of isolated dormant tumor cells, but a lower incidence of microdormant and macrodormant lesions (Fig. 3f). Combined with qRT–PCR results, the expression of *NR2F1*, *DEC2*, *p21* and *p27* was significantly reduced after cogrowth with CAFs (Fig. 3g), suggesting that CAFs have a reactivating effect on dormant SACC-LM cells. Interestingly, the IHC score of LC3 in the CAFs group was remarkably decreased (Fig. 3h) and Ki-67-positive cells were increased (Fig. 3i), suggesting that dormant SACC cells were reactivated after cogrowing with CAFs. As a result, these cells that escaped dormancy were no longer in a state of autophagy activation.

### EVs in the CAFs-CM significantly promoted the reactivation and metastatic recurrence of dormant SACC cells

**Fig. 4:**
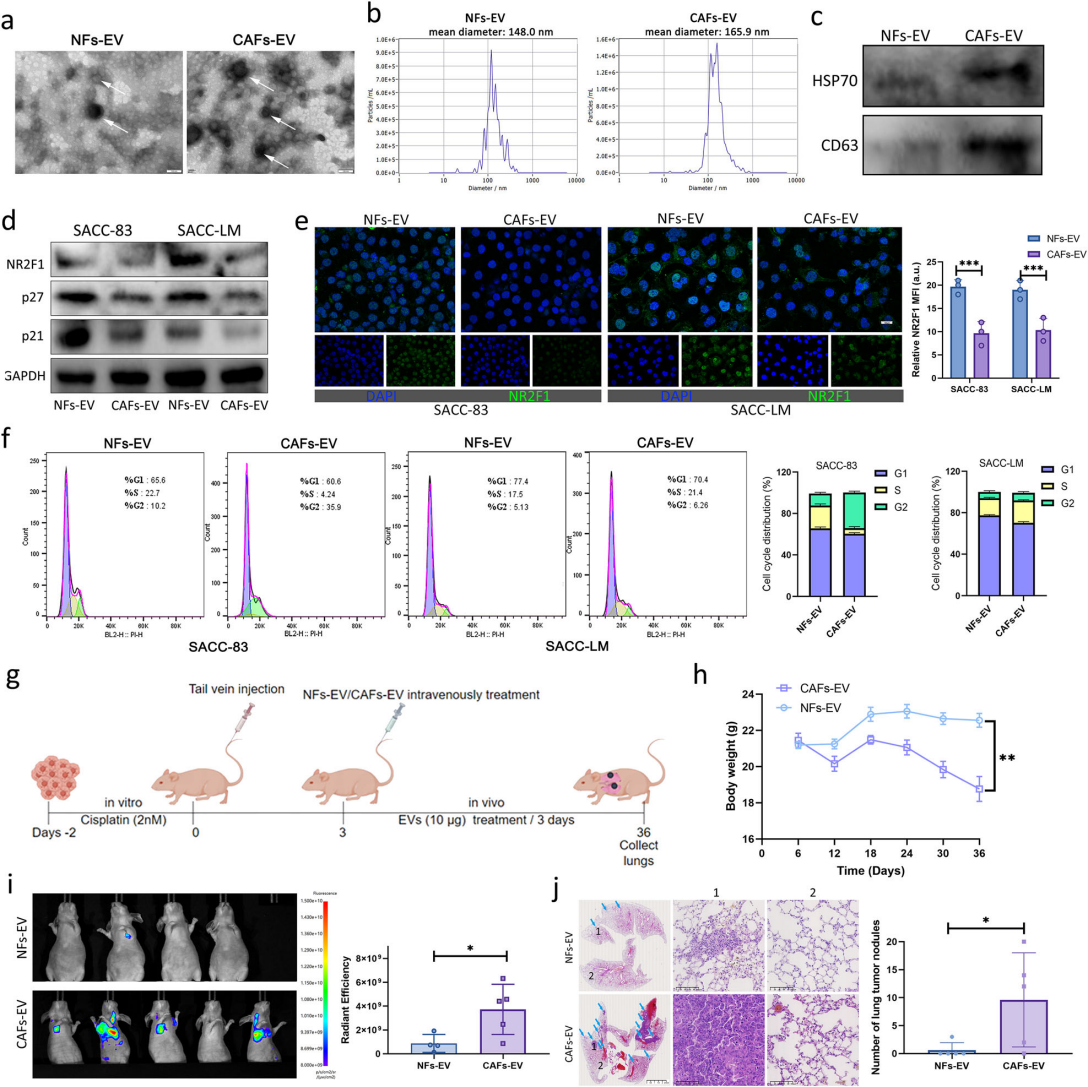
EVs in the CAFs-CM significantly promoted the reactivation and metastatic recurrence of dormant SACC cells. **a** EVs released by NFs and CAFs were detected by TEM. Scale bar, 100 nm. **b** The size range of EVs from NFs and CAFs was detected by NTA. **c** Western blot assay of indicated proteins in EVs. **d**–**f** Western blot assay (**d**), IF assay (**e**) and cell cycle analysis (**f**) of indicated proteins in dormant SACC cells treated with NFs-EVs or CAFs-EVs. Scale bars, 50 μm. **g** Schematic of lung metastasis assay in mice. **h** The body weights of mice treated with NFs-EVs (*n* = 4) or CAFs-EVs (*n* = 5). **i** The lung metastases of mice treated with NFs-EVs or CAFs-EVs were imaged. **j** Representative HE images of lung metastases and statistical analysis of the number of lung metastases.

Because EVs are important in intercellular material transfer, we investigated whether EVs are involved in the communication between CAFs and dormant SACC cells. Therefore, we extracted and purified EVs in NFs-CM and CAFs-CM. NFs-EVs and CAFs-EVs show typical lipid bilayer membrane and disc-shaped structures with a size range of 30–140 nm (Fig. 4a,b). Western blot found the presence of CD63 and HSP70 (positive markers of EVs) in the isolated EVs (Fig. 4c). Interestingly, the number of NFs-EVs was less than that of CAFs-EVs and the diameter was also smaller than that of CAFs-EVs (Fig. 4b). Subsequently, we treated dormant SACC cells with NFs-EVs and CAFs-EVs, respectively. CAFs-EVs downregulated the mRNA levels and abundance of NR2F1, DEC2, p27 and p21 in dormant SACC cells (Fig. 4d,e and Supplementary Fig. 5a). In addition, CAFs-EVs increased the proliferation of dormant SACC cells (Supplementary Fig. 5b). Furthermore, CAFs-EV treatment reduced the proportion of cells in the G0/G1 phase, indicating that CAFs-EVs promoted cell cycle reentry in dormant cells. However, they did not fully reactivate the cells to escape dormancy, probably due to the limitations of the in vitro system (Fig. 4f).

We established a distant metastasis model by injecting cisplatin-induced dormant SACC-LM cells into the tail vein and administered treatments with either CAFs-EVs or NFs-EVs (Fig. 4g). Then, we verified the reactivation effect of CAFs-EVs on dormant SACC cells by analyzing the incidence of SACC lung metastasis recurrence. The body weight of mice in the CAFs-EV treatment group dramatically decreased (Fig. 4h). Importantly, only one out of four mice in the NFs-EV treatment group developed lung metastasis, compared with four out of five mice in the CAFs-EV group (Fig. 4i). Hematoxylin and eosin (HE) section staining also identified that the number of lung metastases in the CAFs-EV treatment group was increased (Fig. 4j). These data demonstrated the important effect of CAFs-EVs in promoting the reactivation of dormant SACC cells.

### Genes specifically upregulated in CAFs

**Fig. 5:**
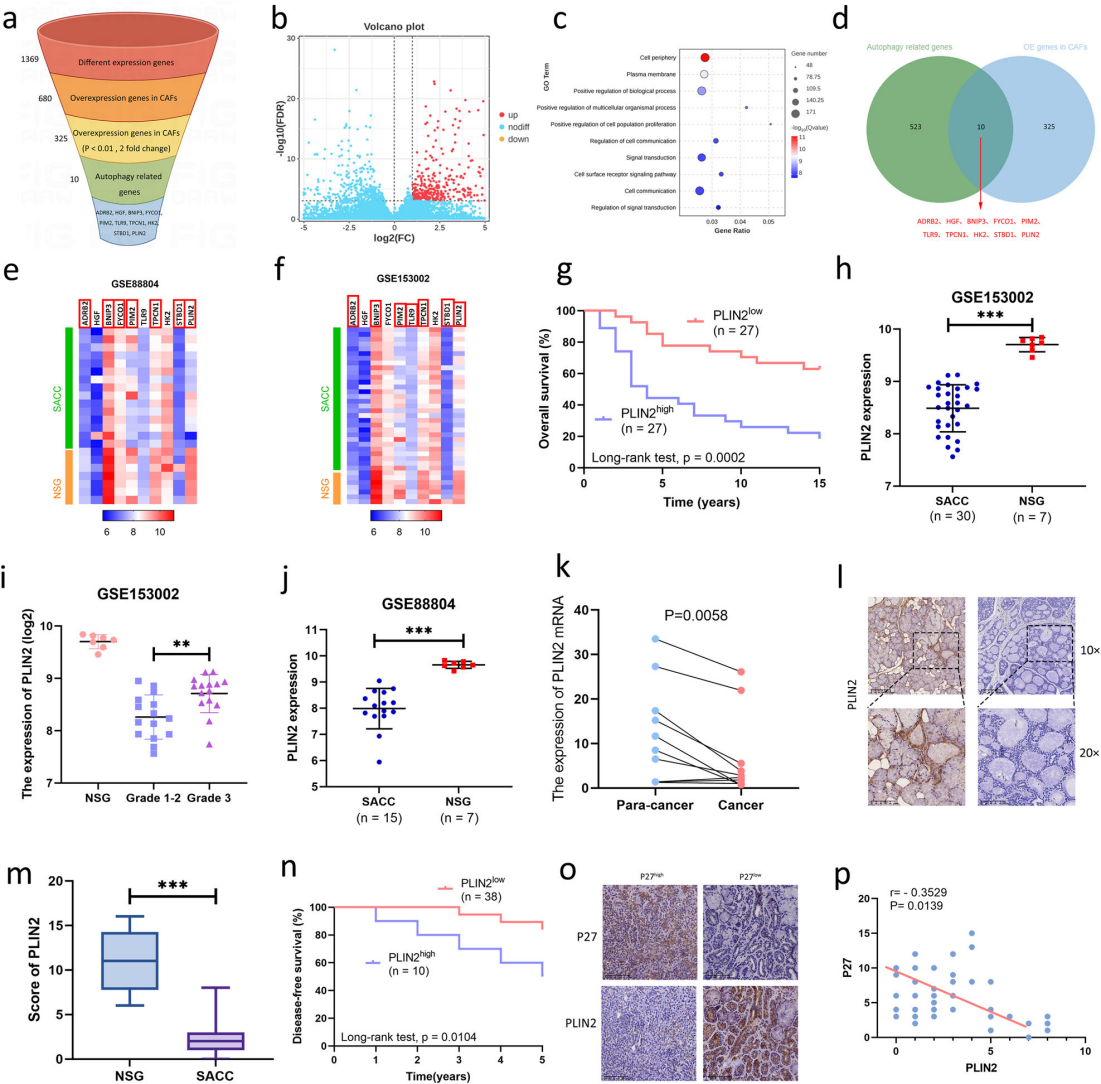
Among the genes specifically overexpressed in CAFs, PLIN2 was found to be expressed at low levels in SACC tissues. **a** The screening process of candidate gene. **b** A total of 325 genes were overexpressed in CAFs. **c** GO enrichment analysis of the genes overexpressed in CAFs. **d** Venn diagram of intersection of overexpressed genes and autophagy-related genes in CAFs. **e**, **f** Expression of candidate genes in the GSE153002 (**e**) and GSE88804 (**f**) datasets. **g** Kaplan–Meier plots of the OS rates in 54 patients with SACC grouped according to high (*n* = 27) and low (*n* = 27) expression of *PLIN2*. **h**–**j** *PLIN2* mRNA expression in SACC and NSG tissues (**h**), in different grades of SACC (**i**) from the GSE153002 dataset, and in the GSE88804 dataset (**j**). **k** qRT–PCR for the *PLIN2* mRNA in ten paired fresh SACC tissues. **l**, **m** IHC analysis of SACC tissues (*n* = 48) and NSG samples (*n* = 10) was performed with the indicated antibodies. Representative staining images (**l**) and the sore of PLIN2 (**m**) are shown. **n** Kaplan–Meier plots of the disease-free survival rates in 48 patients with SACC grouped according to high (*n* = 10) and low (*n* = 38) expression of PLIN2. **o**, **p** IHC analysis of SACC tissues (*n* = 48) was performed with the indicated antibodies (**o**). Correlation analysis of PLIN2 and P27 in SACC tissues is shown (**p**).

Bioinformatics analysis revealed that 680 genes were overexpressed in CAFs compared with NFs. Among the 680 gene, 325 genes with a twofold change (*P* < 0.01) relative to NFs were selected for further screening (Fig. 5a,b). Gene Ontology (GO) enrichment analysis revealed that CAFs also play a necessary role in intercellular signaling, including cell periphery, plasma membrane, positive regulation of biological process, positive regulation of multicellular organismal process, positive regulation of cell population proliferation, regulation of cell communication, signal transduction, cell surface receptor signaling pathway, cell communication and regulation of signal transduction (Fig. 5c). We then intersected the 325 genes highly expressed in CAFs with autophagy-related genes in the Molecular Signatures Database (MSigDB) and screened out ten genes as candidate genes for further research (Fig. 5d).

### PLIN2 was expressed at low levels in SACC tissues and was associated with the clinical prognosis of patients with SACC

The expression levels of the ten candidate genes were evaluated in SACC tissues from the GSE153002 and GSE88804 datasets. Among the ten candidate genes, the expression levels of *ADRB2*, *BNIP3*, *PIM2*, *TPCN1*, *STBD1* and *PLIN2* in SACC tissues were significantly different from those in NSG tissues (Fig. 5e,f and Supplementary Fig. 6a). We also analyzed the relationship between these six genes and patient prognosis using clinical outcome data from a previous study on patients with SACC^22^ (Supplementary Fig. 6b–f). Survival analysis showed that patients with SACC with high *PLIN2* expression had significantly worse 15-year overall survival (OS) (Fig. 5g), and *PLIN2* expression was correlated with the pathological grade of patients with SACC (Fig. 5h–j).

To detect the expression pattern of *PLIN2* mRNA in SACC and NSG tissues, we collected ten pairs of fresh SACC tumor tissues and corresponding adjacent tissues, and extracted total RNA for qRT–PCR verification. As shown in Fig. 5k, the expression of *PLIN2* mRNA in SACC tissues was significantly decreased, suggesting that the expression of PLIN2 showed a downward trend in SACC tissue. Moreover, we investigated the level of PLIN2 in paraffin sections of 48 SACC tumor tissues and 10 NSG tissues samples using IHC staining. IHC scoring results also showed that the PLIN2 level in SACC tissues was significantly decreased (Fig. 5l,m). By analyzing the correlation between PLIN2 levels and clinicopathological factors, the results showed that PLIN2 was significantly correlated with SACC pathological grade and tumor recurrence (Supplementary Table 2). Kaplan–Meier analysis showed that patients with SACC with high PLIN2 expression had a worse prognosis (Fig. 5n). To further characterize the correlation between PLIN2 and tumor dormancy, we also detected the expression level of dormancy-related marker p27 in SACC tumor tissues by IHC and found that the level of PLIN2 in SACC tissues was negatively related to the level of p27 (Fig. 5o,p). In summary, PLIN2 was less expressed in SACC tissues than in NSG tissues, and while PLIN2 expression was upregulated, the clinical prognosis of patients with SACC was worse.

### CAFs-EVs delivering PLIN2 promoted autophagy in dormant SACC cells

**Fig. 6:**
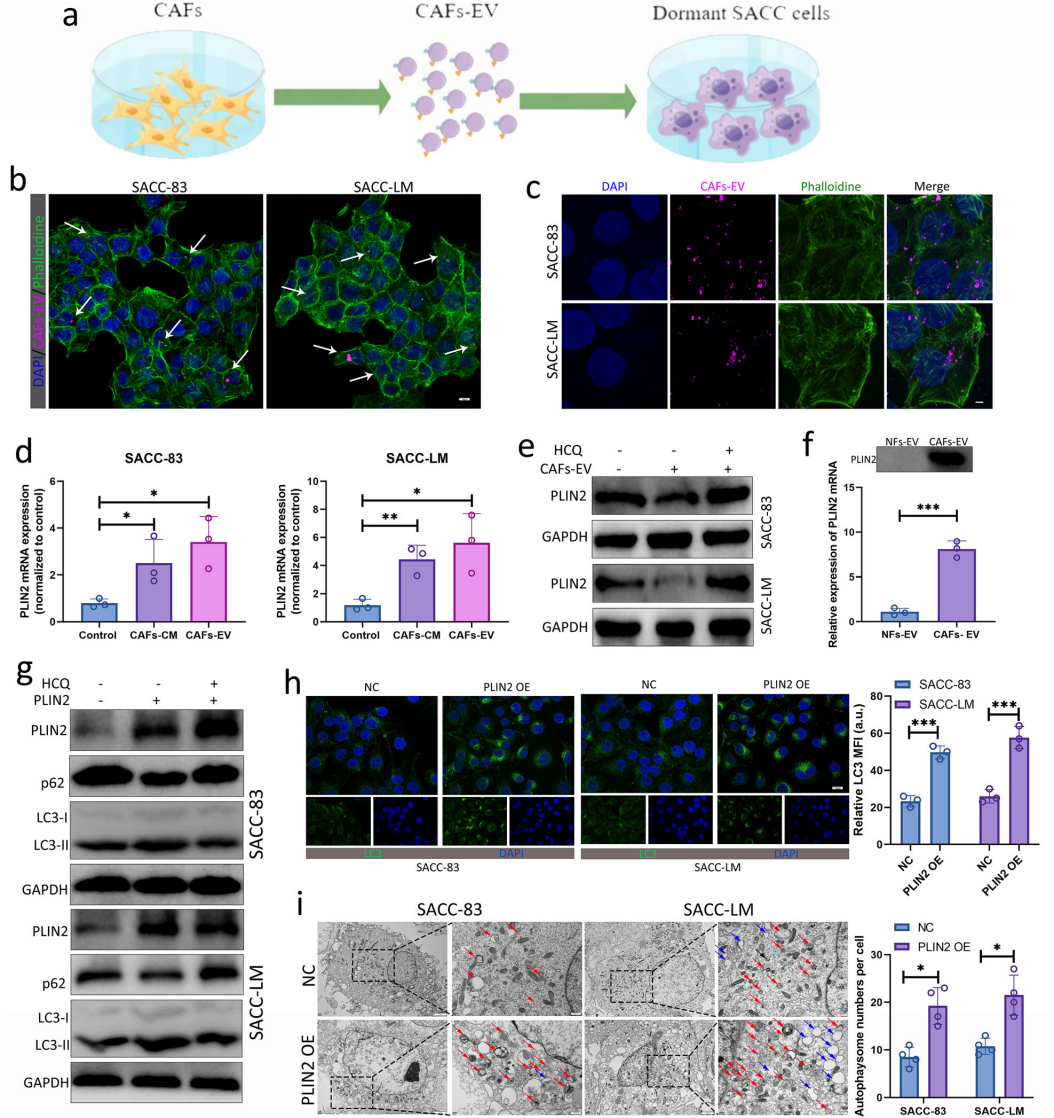
PLIN2 delivered by CAFs-EVs promoted autophagy in dormant SACC cells. **a**–**c** Internalization of DiI-labeled EVs by dormant SACC cells detected via immunofluorescence. Schematic diagram (**a**). Cells treated for 24 h; scale bar, 50 μm (**b**). Cells treated for 8 h; scale bar, 20 μm (**c**). **d** qRT–PCR for the *PLIN2* mRNA in dormant SACC cells treated with CAFs-CM or CAFs-EVs. **e** Western blot for PLIN2 in dormant SACC cells treated with CAFs-EVs or combined with HCQ. **f** Western blot and qRT–PCR for the PLIN2 in NFs-EVs and CAFs-EVs. **g**–**i** Western blot (**g**) for the indicated proteins, IF (**h**) for the LC3 and TEM (**i**) for the autophagosome in dormant SACC cells transfected with *PLIN2* overexpression plasmid. Red arrows indicate autophagosomes; blue arrows indicate the ER.

To verify that CAFs-EVs can be delivered to dormant SACC cells and taken up and utilized by dormant SACC cells, Dio fluorescent dye-labeled CAFs-EVs were cocultured with dormant SACC cells. Using Dio fluorescent dye labeling and laser confocal microscopy, we observed that CAFs-EVs were internalized by dormant SACC cells (Fig. 6a–c). To determine whether PLIN2 in CAFs-EVs could be delivered into SACC cells, we detected the expression of *PLIN2* mRNA in dormant SACC-83 and SACC-LM cells upon CAFs-EV or CAFs-CM treatment. We found that the *PLIN2* mRNA in dormant SACC cells was remarkably increased only when dormant SACC cells were treated with CAFs-CM or CAFs-EVs (Fig. 6d), which indirectly suggested the presence of *PLIN2* mRNA in CAFs-CM or CAFs-EVs. We also examined the levels of PLIN2 in dormant SACC cells after treatment with CAFs-EVs. Interestingly, in the PLIN2 abundance in dormant SACC cells after CAFs-EV treatment had no significant difference, and there was even a trend of decreased expression levels (Fig. 6e). However, when dormant SACC cells were treated with both CAFs-EVs and HCQ, PLIN2 levels were restored, suggesting that CAFs-EVs-delivered PLIN2 may play a role in regulating autophagy in SACC cells. To find direct evidence that PLIN2 existed in CAFs-EVs, we also extracted total RNA from NFs-EVs and CAFs-EVs, and qRT–PCR results showed that the *PLIN2* mRNA in CAFs-EVs was dramatically increased (Fig. 6f). Western blot results showed that CAFs-EVs expressed PLIN2, while NFs-EVs did not express PLIN2 (Fig. 6f). These results collectively indicated that CAFs-EVs were the main source of PLIN2 in the SACC TME.

We next sought to explore the role of PLIN2 on autophagy in SACC cells and constructed a PLIN2 overexpression plasmid. The *PLIN2* overexpression plasmid significantly upregulated the expression of *PLIN2* mRNA in dormant SACC cells (Supplementary Fig. 7a). We then performed western blotting and IF to analyze changes in the autophagy level of the cells. We observed a significant increase in punctate green fluorescence signals in the cytoplasm of dormant SACC cells overexpressing PLIN2, indicating that PLIN2 promoted autophagy in dormant SACC cells (Fig. 6g,h). In addition, we explored the effect of PLIN2 on autophagy in dormant SACC cells through TEM. PLIN2 significantly promoted the number of autophagosomes in dormant SACC cells, which further confirmed that PLIN2 promoted the autophagy of dormant SACC cells (Fig. 6i). Interestingly, we found significant changes in the morphology of the endoplasmic reticulum (ER), including expansion and vesiculization of the ER in cells overexpressing PLIN2 (Fig. 6i). Based on these findings, we believe that CAFs-EV-encapsulated PLIN2 promoted autophagy of dormant SACC cells.

### PLIN2-mediated autophagy promoted the restoration of proliferation and reactivation in dormant SACC cells

**Fig. 7:**
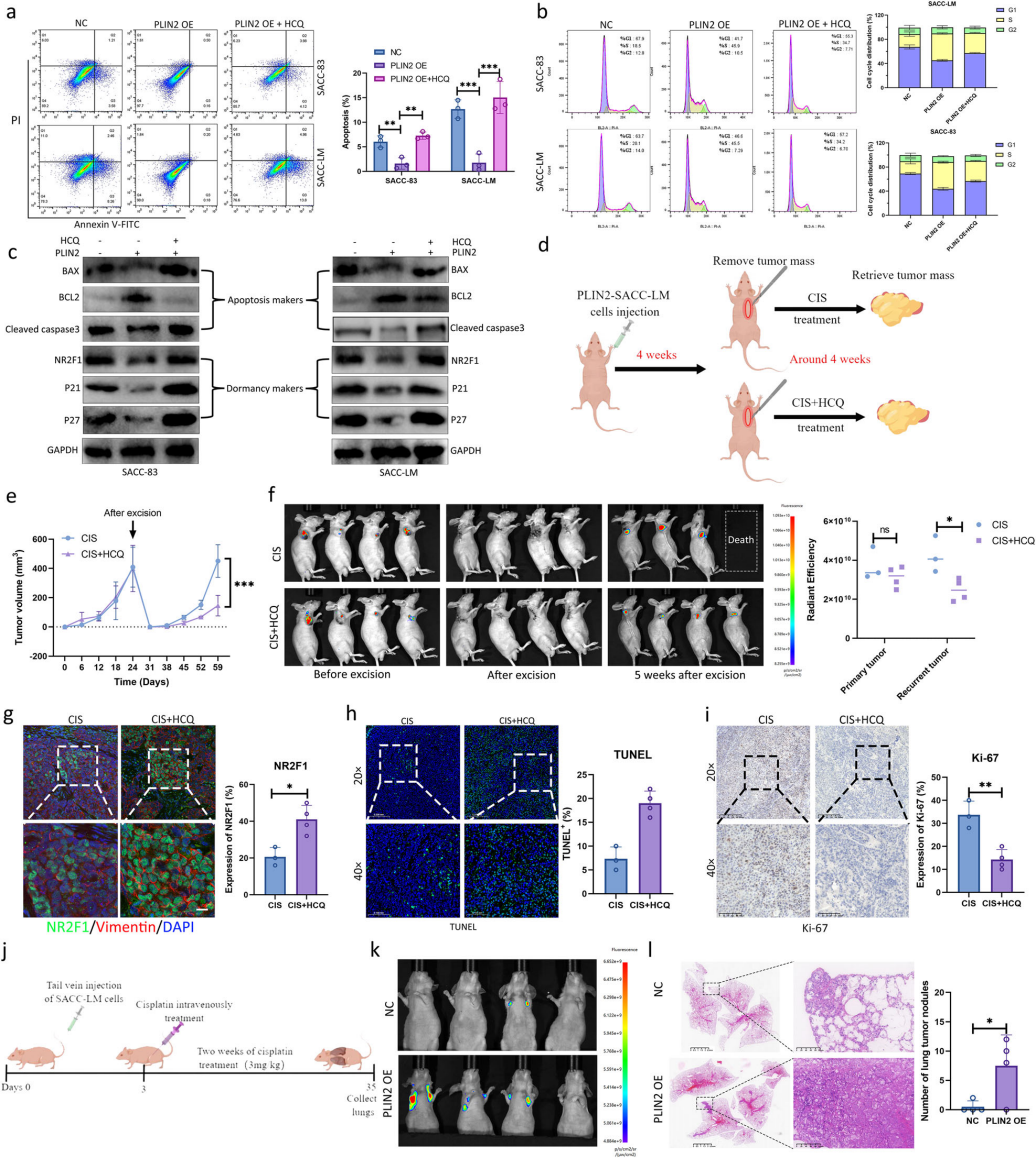
PLIN2-mediated autophagy promoted the restoration of proliferation and reactivation in dormant SACC cells. **a**–**c** Apoptosis analysis (**a**), cell cycle analysis (**b**) and western blot (**c**) of dormant SACC cells transfected with PLIN2 OE or combined with HCQ. **d** Schematic diagram of tumor recurrence experiment in mice. **e** Measurement of the primary tumor volumes and recurrent tumor volumes after excision (*n* = 4 per group). **f** Imaging of the primary tumor and recurrent tumor of nude mice. **g**–**i** IF analysis for NR2F1 (**g**), TUNEL analysis (**h**) and IHC analysis for Ki-67 (**i**) in recurrent tumor tissues. **j** Schematic of the lung metastasis assay in nude mice. **k** Imaging of the lung metastases of nude mice (*n* = 4 per group). **l** Representative HE images of lung metastases and statistical analysis of the number of lung metastases.

Next, we investigated whether PLIN2-mediated autophagy is involved in the reactivation of dormant SACC cells. As shown in Fig. 7a, PLIN2 dramatically reduced the apoptosis in dormant SACC cells, but autophagy inhibition significantly increased the apoptosis. Subsequent cell cycle analysis of dormant SACC cells revealed that PLIN2 significantly decreased the proportion of cells in the G0/G1 phase and increased the proportion in the S phase. However, autophagy inhibition exacerbated cell cycle arrest, suggesting that PLIN2 promoted the reactivation of dormant SACC cells by regulating autophagy (Fig. 7b). In addition, western blot analysis showed that PLIN2 markedly reduced the protein levels of BAX and cleaved caspase-3 in dormant SACC cells, while the levels of BCL2 were increased. However, autophagy inhibition correspondingly restored the protein abundance of BAX, BCL2 and cleaved caspase-3 in dormant SACC cells (Fig. 7c). In agreement with changes in apoptosis-related proteins, PLIN2 reduced the levels of dormancy-related proteins p21, p27 and NR2F1. However, after inhibiting autophagy, the expression levels of p21, p27 and NR2F1 were restored (Fig. 7c). These results suggest that PLIN2 may facilitate the escape of dormant SACC cells from dormancy by regulating autophagy.

Next, a tumor recurrence model was established to investigate whether PLIN2-mediated autophagy promotes the recurrence of SACC. Therefore, we subcutaneously injected SACC-LM cells overexpressing PLIN2 into mice (Supplementary Fig. 7b), until the primary tumor reached approximately 500 mm^3^. After surgical resection, the mice were randomly divided into two groups and were given different treatment methods for 2 weeks: cisplatin (3 mg/kg) treatment group and cisplatin (3 mg/kg) + HCQ (20 mg/kg) combined medication group (Fig. 7d). All mice in the cisplatin treatment group developed local recurrence at the surgical edge, and a mouse died in the third week after surgery. Residual dormant cells persisted at the surgical edge, and reactivation of dormant SACC cells exacerbated tumor recurrence. By contrast, although the combined treatment group of cisplatin and HCQ also experienced tumor recurrence, the size and growth rate of the recurrent tumors were dramatically decreased (Fig. 7e,f and Supplementary Fig. 7c). Consistent with the in vitro findings, histological analysis showed a significant increase in NR2F1-positive cells, along with decreased Ki-67-positive cells and increased TUNEL-positive cells in autophagy-inhibited tumors during cisplatin treatment, suggesting that PLIN2-mediated autophagy accelerates the awakening of dormant SACC cells (Fig. 7g–i and Supplementary Fig. 7d,e). Therefore, our data indicated that PLIN2 promoted the reactivation of local residual dormant SACC cells after primary tumor treatment by upregulating autophagy, ultimately leading to tumor recurrence.

In addition, to explore the role of PLIN2 on distant metastasis of SACC cells, we established a mouse lung metastasis model. After injection of Negative control (NC) or PLIN2-overexpressing SACC-LM cells, cisplatin chemotherapy was performed for 2 weeks (Fig. 7j). We observed that only one mouse in the NC group developed lung metastasis (lung metastasis rate 25%), while three mice in the PLIN2 group developed lung metastasis (lung metastasis rate 75%; Fig. 7k), and the number of lung metastases was remarkably increased in the PLIN2 group (Fig. 7l), suggesting that PLIN2 promoted the recurrence of distant metastasis after SACC chemotherapy.

### The mechanism of PLIN2 regulating autophagy in dormant SACC cells

**Fig. 8:**
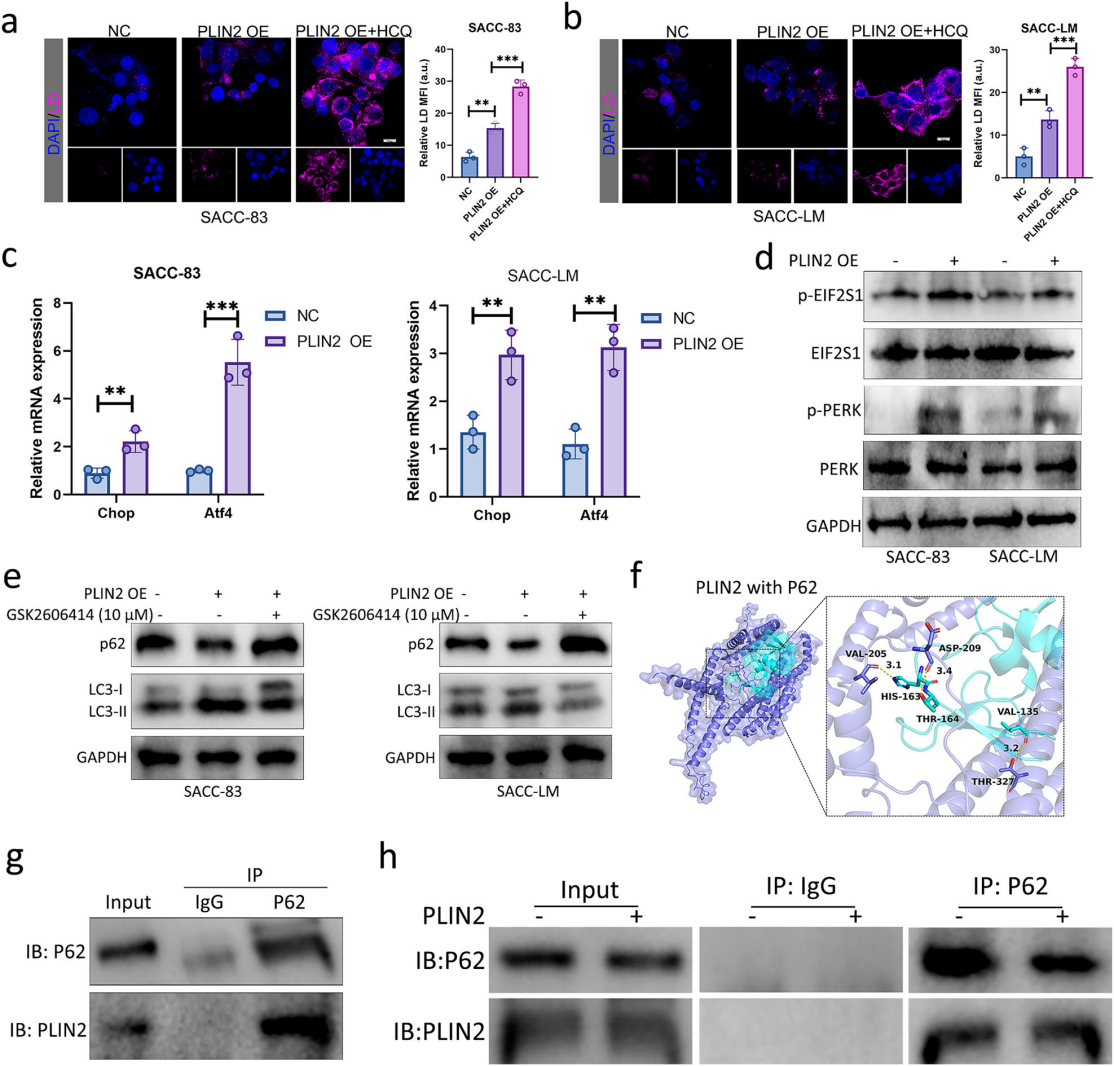
The mechanism of PLIN2 regulating autophagy in dormant SACC cells. **a**,**b** Representative Nile Red staining images of dormant SACC cells overexpressing PLIN2 in the absence or presence of HCQ treatment. Scale bar, 20 μm. **c** qRT–PCR analysis of dormant SACC cells transfected with a NC or PLIN2-overexpressing vector to measure ER stress-related gene expression. **d** Western blot analysis of p-EIF2S1, EIF2S1, p-PERK and PERK levels in dormant SACC cells. **e** The dormant SACC cells were incubated with PERK inhibitor GSK2606414 (10 μM). Then, the protein levels were determined by western blot. **f** Molecular modeling analysis of the interaction between PLIN2 and the binding site within the p62 domain. **g**,**h** Co-IP of p62, followed by western blot for p62 and PLIN2, in dormant SACC-LM cells (**g**) and SACC-LM cells overexpressing PLIN2 (**h**).

Because PLIN2 is involved in the synthesis of LDs, we detected the effect of *PLIN2* on the quantity of LDs through Nile Red staining. As shown in Fig. 8a,b, *PLIN2* promoted the synthesis of LDs in dormant SACC cells, and LDs in dormant SACC cells further increased after autophagy inhibited by HCQ, indicating that PLIN2 may provide energy for dormant SACC cells by promoting lipophagy, thereby contributing to the survival and reactivation of dormant SACC cells. Due to the observation of overexpression of PLIN2 causing morphological changes in the ER by TEM, we speculated that PLIN2 may mediate autophagy through the ER stress pathway in dormant SACC cells. qRT–PCR showed that PLIN2 upregulated *CHOP* and *Atf4* expression (Fig. 8c), and western blot revealed that PLIN2 promoted the phosphorylation of EIF2S1 and PERK (Fig. 8d). Furthermore, PERK (GSK2606414) inhibitor was utilized to further confirm that the PLIN2-mediated ER pathway participated in the activation of autophagy through the PERK signaling pathway. The data revealed that GSK2606414 inhibited PLIN2-mediated LC3B-I/II conversion and reduced P62 expression in dormant SACC cells overexpressing PLIN2 (Fig. 8e), suggesting that PLIN2 mediated the ER stress pathway by directly regulating the PERK signaling pathway and thereby initiated the initial stage of autophagy. To further study the effect of PLIN2 on the degradation stage of autophagy, we first investigated the possible interaction between the cargo protein p62 domain and PLIN2 by molecular docking analysis. In the intermolecular interaction, multiple hydrogen bonds were formed between PLIN2 and p62, such as the binding of VAL205 of PLIN2 and HIS163 of p62 (Fig. 8f). Furthermore, we characterized the p62–PLIN2 interaction by co-IP, and results confirmed the binding of PLIN2 and p62 in dormant SACC-LM cells, suggesting that PLIN2 transported LDs to autophagic lysosomes for degradation through direct interaction with p62 (Fig. 8g,h). In summary, PLIN2 promoted the initial stage of autophagy in dormant SACC cells through the ER stress pathway and directly bound to p62 to transport LDs to lysosomes for lipophagy, thereby providing energy for dormant SACC cells to maintain their survival and promote reactivation.

### Tozasertib showed potential as a PLIN2-targeted drug for CAFs in SACC

**Fig. 9:**
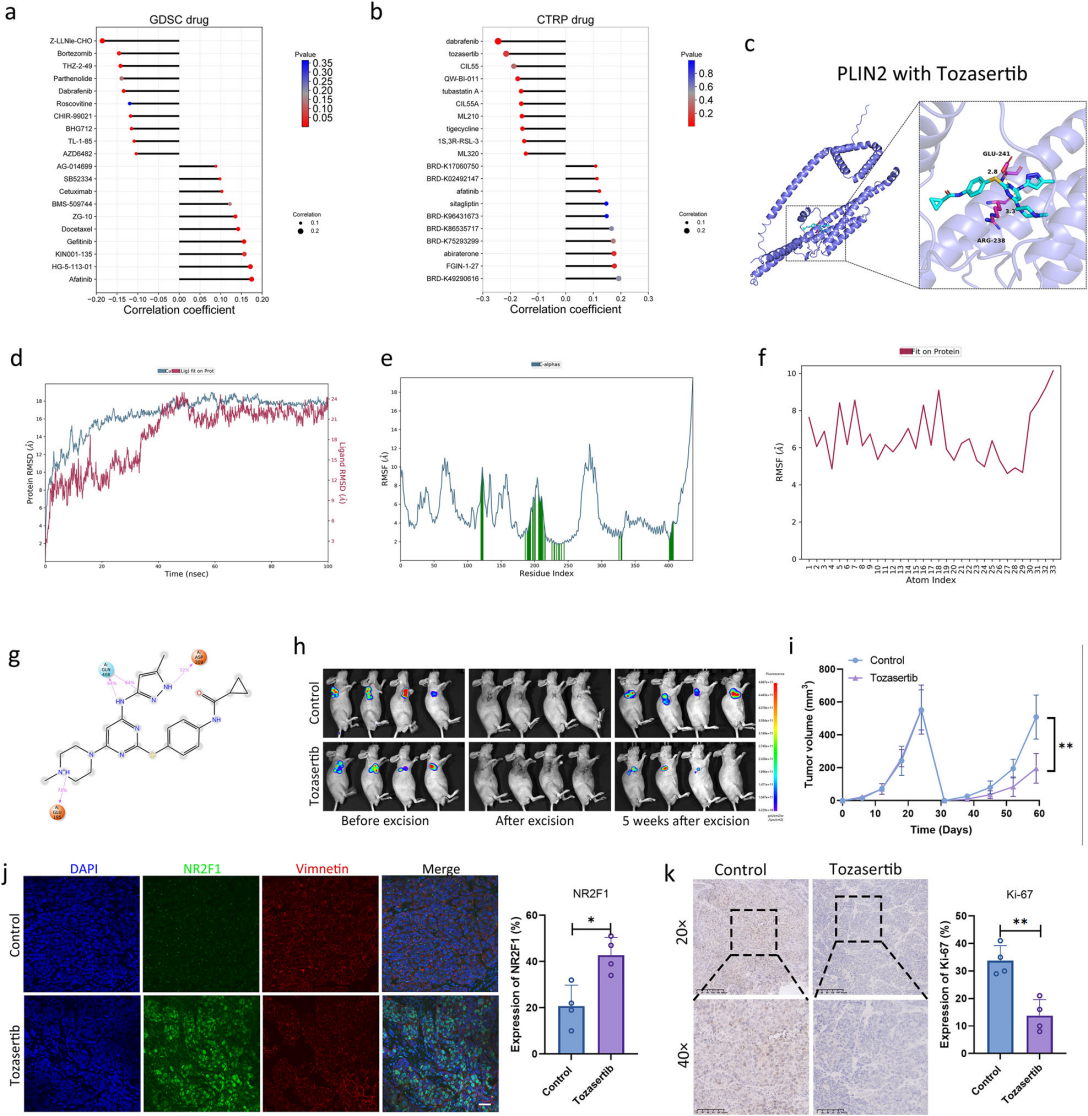
Tozasertib has the potential to serve as a PLIN2-targeted drug for CAFs in SACC. **a**, **b** Study of the sensitivity of anticancer drugs related to PLIN2 through the GDSC (**a**) and CTRP (**b**) databases. **c** The interaction analysis of PLIN2 and tozasertib. **d**–**g** Analysis of tozasertib, including RMSD (**d**), RMSF and visualization of receptor (**e**)–ligand (**f**) interactions (**g**). **h** The primary tumor and recurrent tumor of nude mice were imaged. **i** Measurement of the recurrent tumor volumes (*n* = 4 per group). **j**,**k** IF analysis for NR2F1 (**j**) and IHC analysis for Ki-67 (**k**) in recurrent tumor tissues.

According to the Genomics of Drug Sensitivity in Cancer (GDSC) and Cancer Therapeutics Response Portal (CTRP) databases, we selected the top four drugs negatively correlated with PLIN2, namely Z-LLNle-CHO, tozasertib, dabrafenib and CIL55 (Fig. 9a,b). The interactions between PLIN2 and each ligand were analyzed. The binding energies were −5.5 kcal/mol for PLIN2–Z-LLNle-CHO, −5.4 kcal/mol for PLIN2–CIL55, −7.7 kcal/mol for PLIN2–tozasertib and −6.6 kcal/mol for PLIN2–dabrafenib, indicating stable binding in all cases (Fig. 9c and Supplementary Fig. 8a–c). Subsequently, tozasertib and PLIN2 were selected for molecular dynamics simulations, during which the Root Mean Square Deviation (RMSD) remained relatively stable, settling at approximately 16–17 Å. By contrast, the ligand initially fluctuated but eventually stabilized with an RMSD of around 22 Å, suggesting notable initial conformational changes in both entities and moderate stability of their starting conformations (Fig. 9d). In addition, the amino acid residues involved in small-molecule interactions and the overall Root Mean Square Fluctuation (RMSF) values of the small-molecule atoms were relatively high, suggesting that a more stable binding conformation was achieved compared with their initial states (Fig. 9e,f). During the simulation, PLIN2 and the small molecule formed several interactions, including hydrogen bonds involving GLN408, which occurred with a frequency of 64%. This highlights the crucial role of GLN408 in the binding process (Fig. 9g and Supplementary Fig. 8d,e). In addition, other hydrogen bonds and water bridges were also formed to facilitate their interaction.

To verify the significance of tozasertib in SACC in a recurrence model, SACC-LM cells overexpressing PLIN2 were injected into mice. After surgical resection, the mice were subsequently treated with PBS or tozasertib according to the protocol shown in Supplementary Fig. 8f. Tozasertib treatment significantly reduced the growth of recurrent tumors, which suggests that tozasertib plays a role in the tumor recurrence of SACC (Fig. 9h,i and Supplementary Fig. 8g–i). Moreover, IF showed that the number of NR2F1^+^ dormant cells in mice treated with tozasertib increased significantly (Fig. 9j), while the positive rate of Ki-67 in tumor cells decreased (Fig. 9k). These results suggest that tozasertib has the potential to serve as a PLIN2-targeted therapy in SACC and may play a critical role in preventing tumor recurrence.

## Discussion

**Fig. 10:**
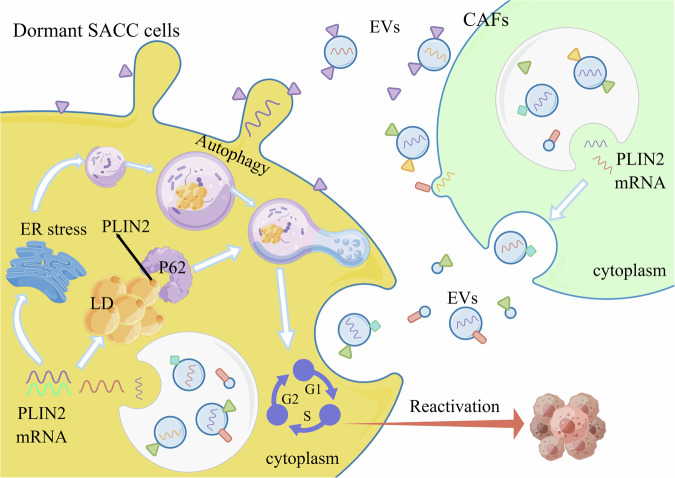
A schematic model of the regulation of LD autophagy by CAFs-EV-derived PLIN2 during SACC dormancy and reactivation. CAF-derived PLIN2 promotes lipophagy of dormant SACC cells to induce the transition of tumor cells from dormancy to reactivation, leading to tumor recurrence and metastasis.

Autophagy plays a crucial role in inducing tumor cells to enter dormancy, promoting the survival of dormant tumor cells and promoting the conversion of dormant tumor cells to proliferation and escape from dormancy^28–31^. In this study (Fig. 10), we found that SACC cells activated autophagy as a means of maintaining their survival after entering a dormant state. In addition, after dormant SACC cells escaped from dormancy, autophagy of tumor cells was subsequently downregulated. Importantly, CAFs in TME further promoted autophagy of dormant SACC cells to induce the transition of tumor cells from dormancy to reactivation. Mechanically, the exosomes secreted by CAFs transported PLIN2 to dormant SACC cells, which initiated autophagy through the ER stress signaling pathway and caused LDs synthesized by PLIN2 to be degraded by binding to the cargo protein p62.

Dormant tumor cells adapt to new TME by activating autophagy and acquiring gene mutations, thereby becoming resistant to antitumor treatments and immune surveillance^32^. Research over the past decade has shown that autophagy is involved in various stages of cancer formation and progression^33^. In tumor dormancy, autophagy can provide dormant tumor cells with sufficient amino acids, ATP and other important substances to meet the energy needs of tumor cells for survival^34^. In our study, the autophagy was activated in dormant SACC cells induced by low-dose cisplatin, but autophagy was subsequently downregulated after tumor cells escaped dormancy. This interesting finding prompted us to explore the significance of autophagy in dormant SACC cells. Consistent with our previous study that autophagy promoted tumor cell survival in hypoxic TME^19^, autophagy promoted the survival ability and number of dormant SACC cells, and inhibition of autophagy severely impaired the survival rate of dormant SACC cells in vitro. Previous study reported that autophagy, as an alternative metabolic pathway, can generate sufficient energy, and autophagy inhibition can impede the survival dormant D2.0R cells^8^. Therefore, combined with our evidence that autophagy contributes to the survival of dormant SACC cells, we speculate that, although dormant SACC cells do not initiate a proliferation program, they remain metabolically active and require almost no exogenous nutrients to survive.

Tumor dormancy has been proven to be the main cause of tumor recurrence and metastasis because dormancy is a reversible process^35,36^. Even decades after tumor treatment, dormant tumor cells under favorable TME can also reawaken and resume proliferation, leading to recurrence and distant metastasis. Activated CAFs, as the most abundant cellular component in TME, can assimilate tumor cells and crosstalk with each other, promoting the malignant behavior of tumor cells by changing extracellular matrix and paracrine patterns. In cholangiocarcinoma, CAFs crosstalk with cancer cells in the TME mainly through the secretion of cytokines and growth factors, extracellular vesicles and extracellular matrix proteins^37,38^. Our vitro experiments confirmed that coculture of CAFs and dormant SACC cells further promoted autophagy of tumor cells, which in turn helped dormant SACC cells restore their proliferation, migration and invasion abilities. However, in vivo experiments showed that the level of autophagy in mice tumor tissues grown together with CAFs was significantly downregulated, suggesting that SACC cells have completely escaped dormancy. The differences in autophagy observed between in vivo and in vitro experiments were attributed to the fact that dormant tumor cells have sufficient time to escape dormancy in nude mice. Autophagy in dormant SACC cells is sufficient to maintain their survival but is not enough to drive their transition to a proliferative state. Intervention by CAFs further enhanced autophagy in dormant SACC cells, facilitating their transition to proliferative reactivation. This result was also confirmed in previous studies, where CAFs induced an increase in autophagy in irradiated lung cancer cells by secreted IGF2 and promoted the autophagy level of liver cancer cells^39,40^. Collectively, autophagy plays a ‘switch’ role in the process of transition from dormancy to proliferation and complete reactivation of tumor cells. We speculate that there is a transitional stage in which SACC cells resume their proliferation program from dormancy to reactivation.

Dormant tumor cells in the G0/G1 phase could re-enter the cell cycle after receiving signals of proliferation. Extracellular vesicles secreted by CAFs serve as an exogenous signal and have been reported to have different effects on the malignant progression of different types of tumor^41,42^. In our study, CAFs-EVs were shown to promote the re-entry of dormant SACC cells into proliferation, reduce the proportion of cells in the G0/G1 phase and thereby facilitate distant colonization and metastasis. This is supported by a report in breast cancer, where metabolically dormant cells treated with CAF-derived mtDNA-EVs were able to escape dormancy and give rise to hormone-therapy-resistant metastatic tumors^14^. Extracellular vesicles not only facilitate the reactivation of dormant tumor cells, but also promote tumor cells to enter a dormant state. FFor example, extracellular vesicles released by mesenchymal stem cells containing miR-222/223 can induce some breast cancer cells to enter a state of dormancy, conferring drug resistance^43^. In short, whether extracellular vesicles secreted by mother cells promote tumor cells to enter dormancy or reactivate dormant tumor cells mainly depends on the molecular information they carry.

CAFs showed phenotypic and functional heterogeneity according to different sources and different TMEs^44^. Similarly, the difference in genetic information carried by CAFs-EVs was also a reflection of their heterogeneity. In our study, we found that CAFs significantly overexpress PLIN2, which is encapsulated in extracellular vesicles and delivered to dormant SACC cells. In recent years, dysregulation of PLIN2 expression has been confirmed in a variety of cancers and is correlated with patient survival, including gastric cancer and renal cell carcinoma^45,46^. However, the expression and role of PLIN2 in SACC still remain unclear. Here, we demonstrated that PLIN2 was differentially expressed in 48 SACC tissues compared with 10 NSG tissues, and PLIN2 may serve as a potential marker of poor prognosis in patients with SACC.

PLIN2 is a member of the PAT family, assists in the storage of lipids in LD and is directly involved in the synthesis of LDs, special fat-storing organelles in cells^47^. Under nutrient scarcity, tumor cells break down LDs via ATGL-mediated lipolysis or autophagy-mediated degradation, subsequently generating energy through fatty acid oxidation. Lipophagy is a selective autophagy that targets LDs and is an important regulatory mechanism for LD catabolism. Our data revealed that overexpression of PLIN2 promoted autophagy and increased LD content, whereas inhibition of autophagy led to a further accumulation of LDs in dormant SACC cells. It was worth noting that PLIN2 promoted the synthesis of LDs after entering dormant SACC cells, and further promoted the autophagy level of dormant SACC cells to degrade LDs to provide energy for tumor cells, thereby helping dormant SACC cells to restore their proliferation state and escape from dormancy. Increasing evidence suggests that autophagy is regulated via the PERK-dependent ER stress response^48^. A recent study showed that TRAF3IP3-mediated ER stress induced protective autophagy by activating the PERK–ATF4–CHOP pathway to regulate the growth of lung adenocarcinoma cells^49^. Mechanistically, PLIN2 induces ER stress by activating the PERK signaling pathway to initiate autophagy in dormant SACC cells, and it physically binds to p62 to deliver LDs to lysosomes for degradation via the autophagy pathway. Furthermore, our results indicate that tozasertib can stably bind to PLIN2 and downregulate its mRNA and protein levels, providing a theoretical basis for its potential use as a PLIN2-targeted therapy in CAFs for the treatment of SACC.

In summary, our data provide compelling evidence for the crucial role of CAFs in SACC dormancy, showing that PLIN2 carried by CAFs-EVs regulates LD autophagy in dormant SACC cells, thereby contributing to tumor recurrence and metastasis. Therefore, targeting PLIN2 for autophagy inhibition as an adjunct to the treatment of SACC may effectively kill dormant tumor cells, prevent SACC recurrence and improve the survival rate of patients with SACC.

## Supplementary information

Supplementary Information

## References

[1] Coca-Pelaz, A. et al. Adenoid cystic carcinoma of the head and neck—an update. *Oral. Oncol.* **51**, 652–661 (2015). 25943783 10.1016/j.oraloncology.2015.04.005

[2] Fang, Y. et al. Current opinions on diagnosis and treatment of adenoid cystic carcinoma. *Oral. Oncol.* **130**, 105945 (2022). 35662026 10.1016/j.oraloncology.2022.105945

[3] Umeda, M. et al. Tumor-doubling time and onset of pulmonary metastasis from adenoid cystic carcinoma of the salivary gland. *Oral Surg. Oral Med. Oral Pathol. Oral Radiol. Endod.* **88**, 473–478 (1999). 10519758 10.1016/s1079-2104(99)70065-x

[4] Kirkland, J. L. Tumor dormancy and disease recurrence. *Cancer Metast. Rev.* **42**, 9–12 (2023). 10.1007/s10555-023-10096-0 PMC9986652 36877312

[5] Jahangiri, L. & Ishola, T. Dormancy in breast cancer, the role of autophagy, lncRNAs, miRNAs and exosomes. *Int. J. Mol. Sci.*10.3390/ijms23095271 (2022). 10.3390/ijms23095271 PMC9105119 35563661

[6] Yang, P. et al. Novel targets for gastric cancer: the tumor microenvironment (TME), *N*^6^-methyladenosine (m^6^A), pyroptosis, autophagy, ferroptosis and cuproptosis. *Biomed. Pharmacother.* **163**, 114883 (2023). 37196545 10.1016/j.biopha.2023.114883

[7] Xi, L. et al. Hypoxia-stimulated ATM activation regulates autophagy-associated exosome release from cancer-associated fibroblasts to promote cancer cell invasion. *J. Extracell. Vesicles* **10**, e12146 (2021). 34545708 10.1002/jev2.12146 PMC8452512

[8] Vera-Ramirez, L., Vodnala, S. K., Nini, R., Hunter, K. W. & Green, J. E. Autophagy promotes the survival of dormant breast cancer cells and metastatic tumour recurrence. *Nat. Commun.* **9**, 1944 (2018). 29789598 10.1038/s41467-018-04070-6 PMC5964069

[9] Biffi, G. & Tuveson, D. A. Diversity and biology of cancer-associated fibroblasts. *Physiol. Rev.* **101**, 147–176 (2021). 32466724 10.1152/physrev.00048.2019 PMC7864232

[10] Zhang, J. et al. Cancer-associated fibroblasts promote the migration and invasion of gastric cancer cells via activating IL-17a/JAK2/STAT3 signaling. *Ann. Transl. Med.* **8**, 877 (2020). 32793721 10.21037/atm-20-4843 PMC7396760

[11] Chhabra, Y. & Weeraratna, A. T. Fibroblasts in cancer: unity in heterogeneity. *Cell* **186**, 1580–1609 (2023). 37059066 10.1016/j.cell.2023.03.016 PMC11422789

[12] Niu, N. et al. Tumor cell-intrinsic epigenetic dysregulation shapes cancer-associated fibroblasts heterogeneity to metabolically support pancreatic cancer. *Cancer cell* **42**, 869–884 (2024). 38579725 10.1016/j.ccell.2024.03.005

[13] Fane, M. E. et al. Stromal changes in the aged lung induce an emergence from melanoma dormancy. *Nature* **606**, 396–405 (2022). 35650435 10.1038/s41586-022-04774-2 PMC9554951

[14] Sansone, P. et al. Packaging and transfer of mitochondrial DNA via exosomes regulate escape from dormancy in hormonal therapy-resistant breast cancer. *Proc. Natl Acad. Sci. USA* **114**, E9066–E9075 (2017). 29073103 10.1073/pnas.1704862114 PMC5664494

[15] Cheng, C., Geng, F., Cheng, X. & Guo, D. Lipid metabolism reprogramming and its potential targets in cancer. *Cancer Commun.* **38**, 27 (2018). 10.1186/s40880-018-0301-4 PMC5993136 29784041

[16] Liu, R. et al. Choline kinase alpha 2 acts as a protein kinase to promote lipolysis of lipid droplets. *Mol. Cell* **81**, 2722–2735 (2021). 34077757 10.1016/j.molcel.2021.05.005

[17] Maan, M., Peters, J. M., Dutta, M. & Patterson, A. D. Lipid metabolism and lipophagy in cancer. *Biochem. Biophys. Res. Commun.* **504**, 582–589 (2018). 29438712 10.1016/j.bbrc.2018.02.097 PMC6086774

[18] Loix, M. et al. Perilipin-2 limits remyelination by preventing lipid droplet degradation. *Cell. Mol. Life Sci.* **79**, 515 (2022). 36100764 10.1007/s00018-022-04547-0 PMC11803036

[19] Gao, L. et al. CircCDR1as upregulates autophagy under hypoxia to promote tumor cell survival via AKT/ERK(½)/mTOR signaling pathways in oral squamous cell carcinomas. *Cell Death Dis.* **10**, 745 (2019). 31582727 10.1038/s41419-019-1971-9 PMC6776509

[20] Dai, L. et al. Hypoxia induced cell dormancy of salivary adenoid cystic carcinoma through miR-922/DEC2 axis. *Transl. Oncol.* **40**, 101868 (2024). 38141378 10.1016/j.tranon.2023.101868 PMC10751830

[21] Pang, X. et al. OSCC cell-secreted exosomal CMTM6 induced M2-like macrophages polarization via ERK1/2 signaling pathway. *Cancer Immunol. Immunother.* **70**, 1015–1029 (2021). 33104837 10.1007/s00262-020-02741-2 PMC10991130

[22] Ferrarotto, R. et al. Proteogenomic analysis of salivary adenoid cystic carcinomas defines molecular subtypes and identifies therapeutic targets. *Clin. Cancer Res.* **27**, 852–864 (2021). 33172898 10.1158/1078-0432.CCR-20-1192 PMC7854509

[23] Jiao, Y. et al. Dormant cancer cells and polyploid giant cancer cells: the roots of cancer recurrence and metastasis. *Clin. Transl. Med.* **14**, e1567 (2024). 38362620 10.1002/ctm2.1567 PMC10870057

[24] Wang, L. et al. Cell-cell contact-driven EphB1 *cis*- and *trans*-signalings regulate cancer stem cells enrichment after chemotherapy. *Cell Death Dis.* **13**, 980 (2022). 36402751 10.1038/s41419-022-05385-5 PMC9675789

[25] Xu, J. et al. Proteomic profiling of extracellular vesicles and particles reveals the cellular response to cisplatin in NSCLC. *Thorac. Cancer* **12**, 2601–2610 (2021). 34520129 10.1111/1759-7714.14147 PMC8487815

[26] Wang, L. et al. Chromatin accessibility regulates chemotherapy-induced dormancy and reactivation. *Mol. Ther. Nucleic Acids* **26**, 269–279 (2021). 34513309 10.1016/j.omtn.2021.07.019 PMC8413835

[27] Chai, C. et al. Single-cell transcriptome analysis of epithelial, immune, and stromal signatures and interactions in human ovarian cancer. *Commun. Biol.* **7**, 131 (2024). 38278958 10.1038/s42003-024-05826-1 PMC10817929

[28] Ovadia, E. M. et al. Understanding ER^+^ breast cancer dormancy using bioinspired synthetic matrices for long-term 3D culture and insights into late recurrence. *Adv. Biosyst.* **4**, e2000119 (2020). 32603024 10.1002/adbi.202000119 PMC7807552

[29] Yu, Z. et al. Blockage of SLC31A1-dependent copper absorption increases pancreatic cancer cell autophagy to resist cell death. *Cell Prolif.* **52**, e12568 (2019). 30706544 10.1111/cpr.12568 PMC6496122

[30] Aqbi, H. F. et al. Autophagy-deficient breast cancer shows early tumor recurrence and escape from dormancy. *Oncotarget* **9**, 22113–22122 (2018). 29774126 10.18632/oncotarget.25197 PMC5955162

[31] Shinde, A. et al. Spleen tyrosine kinase-mediated autophagy is required for epithelial–mesenchymal plasticity and metastasis in breast cancer. *Cancer Res.* **79**, 1831–1843 (2019). 30733195 10.1158/0008-5472.CAN-18-2636 PMC6467765

[32] Sosa, M. S., Bragado, P., Debnath, J. & Aguirre-Ghiso, J. A. Regulation of tumor cell dormancy by tissue microenvironments and autophagy. *Adv. Exp. Med. Biol.* **734**, 73–89 (2013). 23143976 10.1007/978-1-4614-1445-2_5 PMC3651695

[33] Tamamouna, V., Pavlou, E., Neophytou, C. M., Papageorgis, P. & Costeas, P. Regulation of metastatic tumor dormancy and emerging opportunities for therapeutic intervention. *Int. J. Mol. Sci.*10.3390/ijms232213931 (2022). 10.3390/ijms232213931 PMC9698240 36430404

[34] Shimizu, T. et al. IGF2 preserves osteosarcoma cell survival by creating an autophagic state of dormancy that protects cells against chemotherapeutic stress. *Cancer Res.* **74**, 6531–6541 (2014). 25273088 10.1158/0008-5472.CAN-14-0914

[35] Elkholi, I. E., Lalonde, A., Park, M. & Côté, J. F. Breast cancer metastatic dormancy and relapse: an enigma of microenvironment(s). *Cancer Res.* **82**, 4497–4510 (2022). 36214624 10.1158/0008-5472.CAN-22-1902 PMC9755970

[36] Aouad, P., Quinn, H. M., Berger, A. & Brisken, C. Tumor dormancy: EMT beyond invasion and metastasis. *Genes* **62**, e23552 (2024). 10.1002/dvg.23552 37776086

[37] Duangdara, J. et al. CP-673451, a selective platelet-derived growth factor receptor tyrosine kinase inhibitor, induces apoptosis in opisthorchis viverrini-associated cholangiocarcinoma via Nrf2 suppression and enhanced ROS. *Pharmaceuticals*10.3390/ph17010009 (2023). 10.3390/ph17010009 PMC10821224 38275995

[38] Clapéron, A. et al. Hepatic myofibroblasts promote the progression of human cholangiocarcinoma through activation of epidermal growth factor receptor. *Hepatology* **58**, 2001–2011 (2013). 23787814 10.1002/hep.26585

[39] Martinez-Outschoorn, U. E., Lisanti, M. P. & Sotgia, F. Catabolic cancer-associated fibroblasts transfer energy and biomass to anabolic cancer cells, fueling tumor growth. *Semin. Cancer Biol.* **25**, 47–60 (2014). 24486645 10.1016/j.semcancer.2014.01.005

[40] Zhao, Z. et al. Cancer-associated fibroblasts endow stem-like qualities to liver cancer cells by modulating autophagy. *Cancer Manag. Res.* **11**, 5737–5744 (2019). 31296998 10.2147/CMAR.S197634 PMC6598753

[41] Cui, Y. et al. An exosome-derived lncRNA signature identified by machine learning associated with prognosis and biomarkers for immunotherapy in ovarian cancer. *Front. Immunol.* **15**, 1228235 (2024). 38404588 10.3389/fimmu.2024.1228235 PMC10884316

[42] Xu, L. et al. ANXA3-rich exosomes derived from tumor-associated macrophages regulate ferroptosis and lymphatic metastasis of laryngeal squamous cell carcinoma. *Cancer Immunol. Res.* **12**, 614–630 (2024). 38393971 10.1158/2326-6066.CIR-23-0595

[43] Bliss, S. A. et al. Mesenchymal stem cell-derived exosomes stimulate cycling quiescence and early breast cancer dormancy in bone marrow. *Cancer Res.* **76**, 5832–5844 (2016). 27569215 10.1158/0008-5472.CAN-16-1092

[44] Caligiuri, G. & Tuveson, D. A. Activated fibroblasts in cancer: perspectives and challenges. *Cancer Cell* **41**, 434–449 (2023). 36917949 10.1016/j.ccell.2023.02.015 PMC11022589

[45] Sun, X. et al. The modification of ferroptosis and abnormal lipometabolism through overexpression and knockdown of potential prognostic biomarker perilipin2 in gastric carcinoma. *Gastric Cancer* **23**, 241–259 (2020). 31520166 10.1007/s10120-019-01004-z

[46] Cao, Q. et al. Overexpression of PLIN2 is a prognostic marker and attenuates tumor progression in clear cell renal cell carcinoma. *Int. J. Oncol.* **53**, 137–147 (2018). 29749470 10.3892/ijo.2018.4384 PMC5958875

[47] Hayakawa, M. et al. Lipid droplet accumulation and adipophilin expression in follicular thyroid carcinoma. *Biochem. Biophys. Res. Commun.* **640**, 192–201 (2023). 36521425 10.1016/j.bbrc.2022.12.007

[48] Shao, Y., Zheng, L. & Jiang, Y. Cadmium toxicity and autophagy: a review. *Biometals* **37**, 609–629 (2024). 38277035 10.1007/s10534-023-00581-y

[49] Zhao, G. et al. TRAF3IP3 induces ER stress-mediated apoptosis with protective autophagy to inhibit lung adenocarcinoma proliferation. *Adv. Sci.* **12**, e2411020 (2025). 10.1002/advs.202411020 PMC12061266 40068093

