# Supplementary material for: Cancer-associated fibroblast-derived extracellular vesicles regulate lipophagy through PLIN2 to modulate dormancy in salivary gland adenoid cystic carcinoma cells
Source: Exp Mol Med. 2025 Dec 18;57(12):2852–68. doi: 10.1038/s12276-025-01600-3 (PMC12800263; doi:10.1038/s12276-025-01600-3)
Supplement: Supplementary file 1 — Supplementary Information [file 12276_2025_1600_MOESM1_ESM.pdf]

# Supplementary Information

## Supplementary Figures

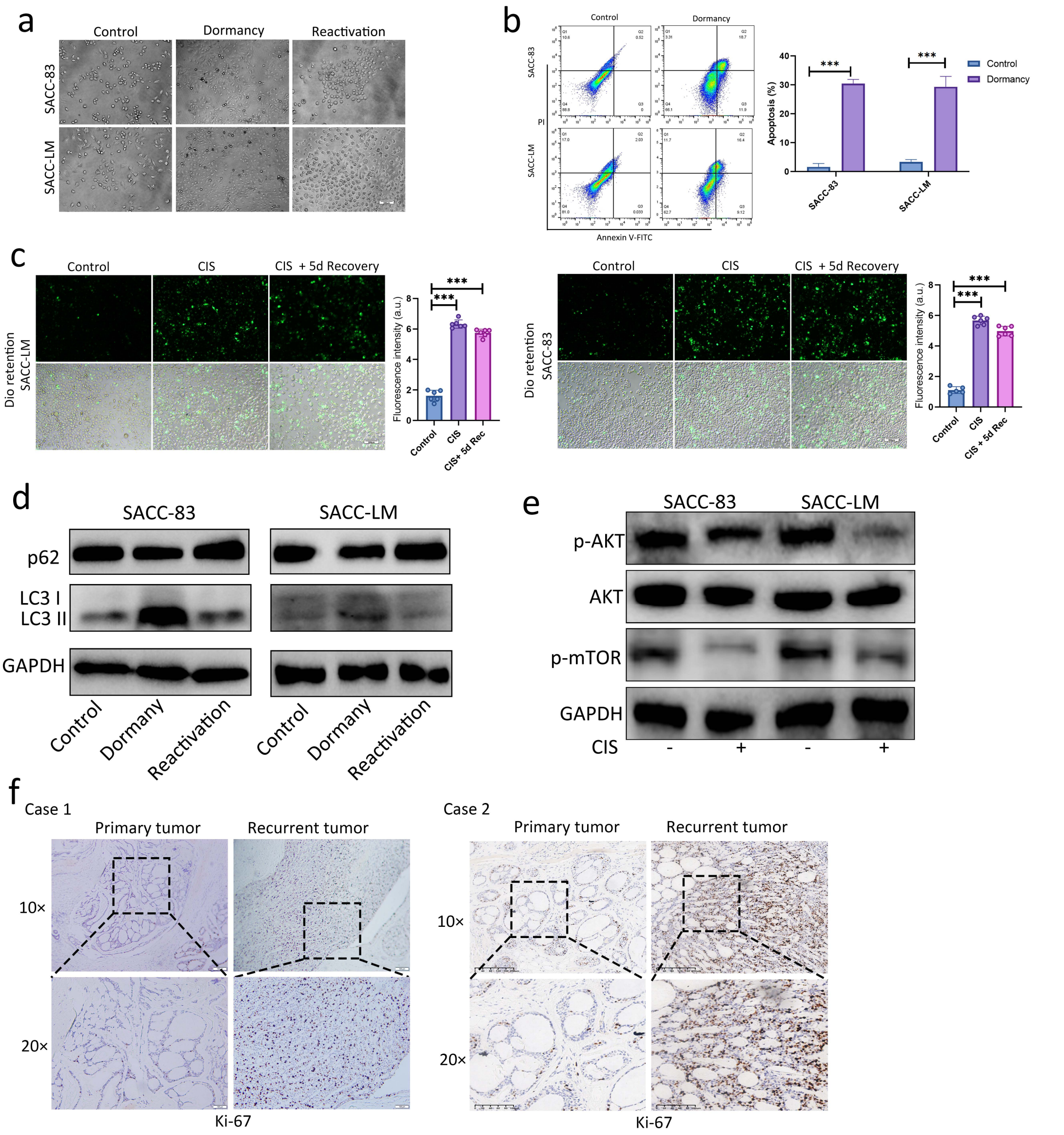


**Supplementary Fig. 1: Dormant SACC tumor cells were autophagic. a** The microscope images of dormant and reactivated SACC cells. **b** Flow cytometry analysis of apoptosis in SACC cells treated with cisplatin. **c** Dio staining analysis of SACC cells treated with cisplatin at different time points. **d** Western blot analysis of proteins obtained from dormant and reactivated SACC cells. **e** Western blot analysis of p-AKT, AKT, p-mTOR levels of SACC cells treated with cisplatin. **f** Assessment of Ki-67 in 6 SACC patients with recurrence after surgery and chemotherapy. Representative images of IHC.

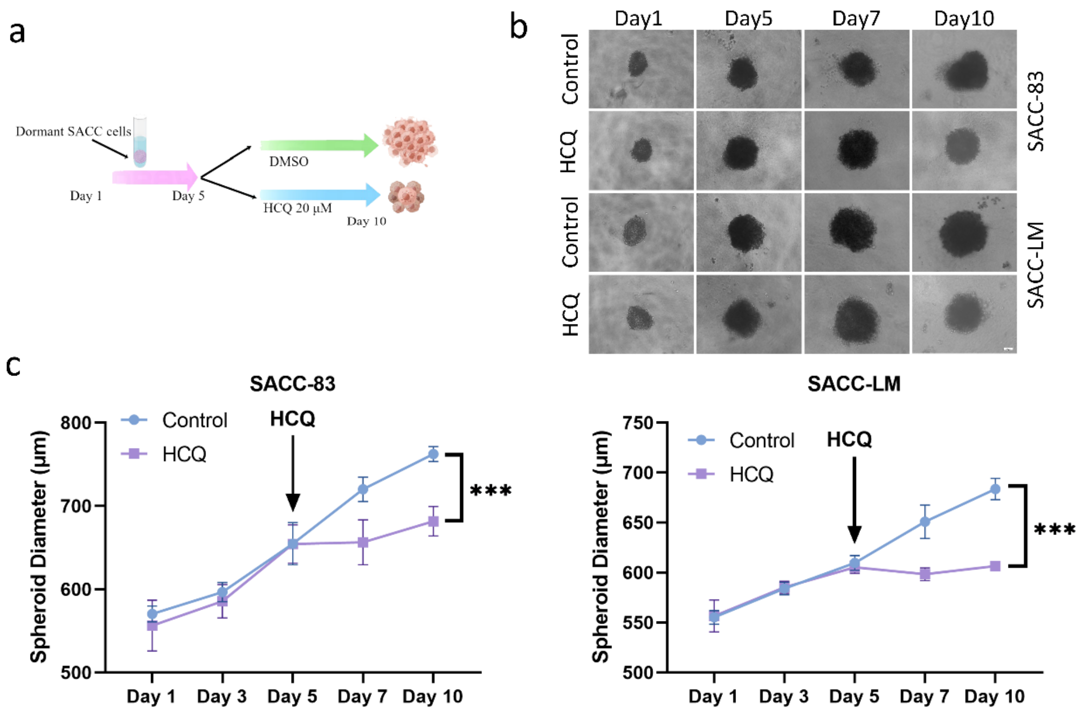


**Supplementary Fig. 2: Autophagy inhibition reduced the sphere forming ability of dormant SACC cells. a** Scheme of sphere formation assay in dormant SACC cells treated with HCQ. **b, c** Sphere formation assay on dormant SACC cells treated with HCQ (b). Scale bar, 200 μm. Statistical analysis of Sphere diameter (c).

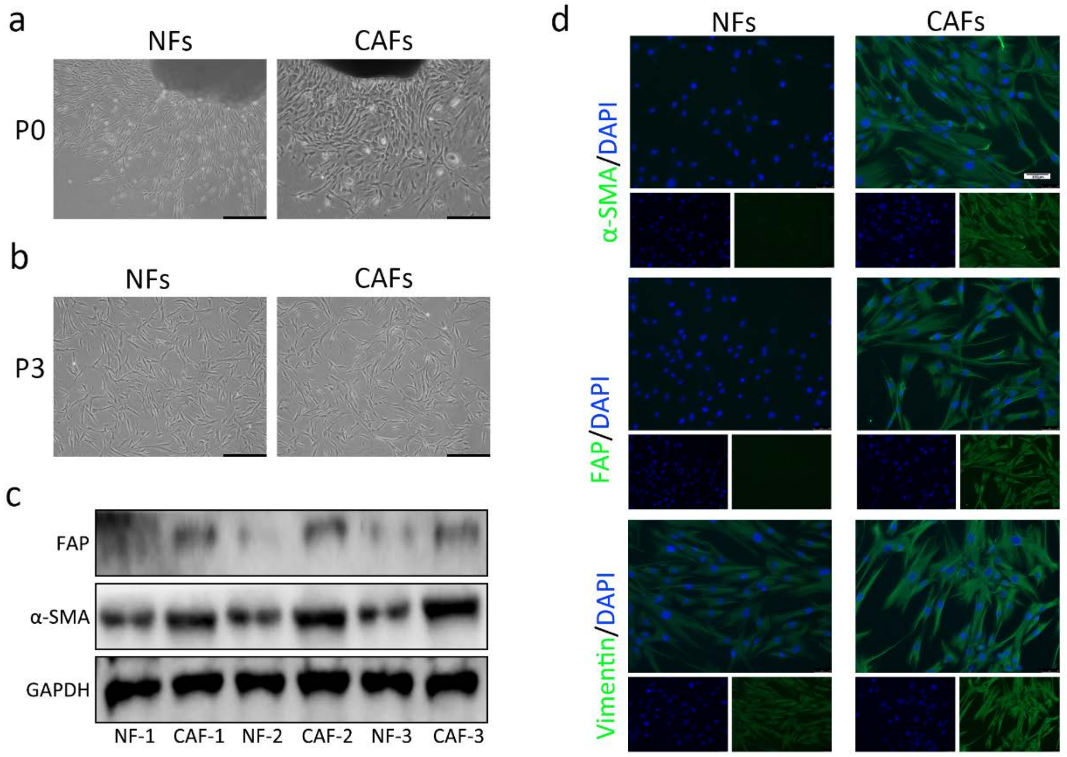

**Supplementary Fig. 3: Identification of NFs and CAFs. a, b** Representative cell morphology images of fibroblasts isolated from NSG and SACC tissues. Scale bar, 200 μm. **c** Western blot of α-SMA, vimentin, and FAP levels in fibroblasts. **d** Immunofluorescence analysis of α-SMA, vimentin, and FAP levels in fibroblasts. Scale bar, 100 μm.

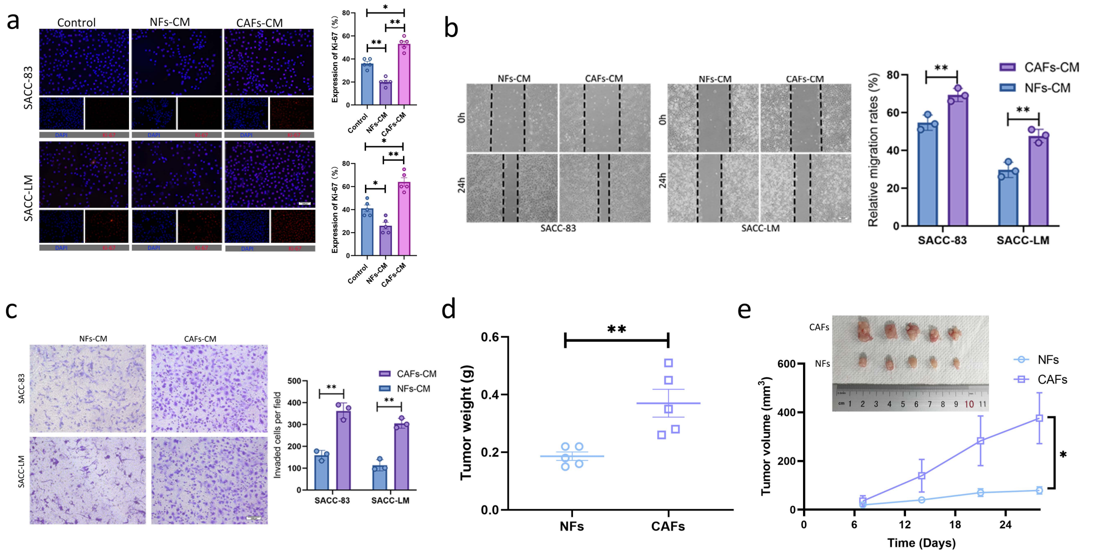


**Supplementary Fig. 4: CAFs promoted the proliferation, migration, invasion and tumorigenic ability of dormant SACC cells. a** Assessment of Ki67 in dormant SACC cells treated with NFs-CM or CAFs-CM. Scale bar, 100 μm. **b, c** Migration and Transwell invasion assays in dormant SACC cells treated with NFs-CM or CAFs-CM. **d, e** The weights and volumes of the tumor were observed and measured.

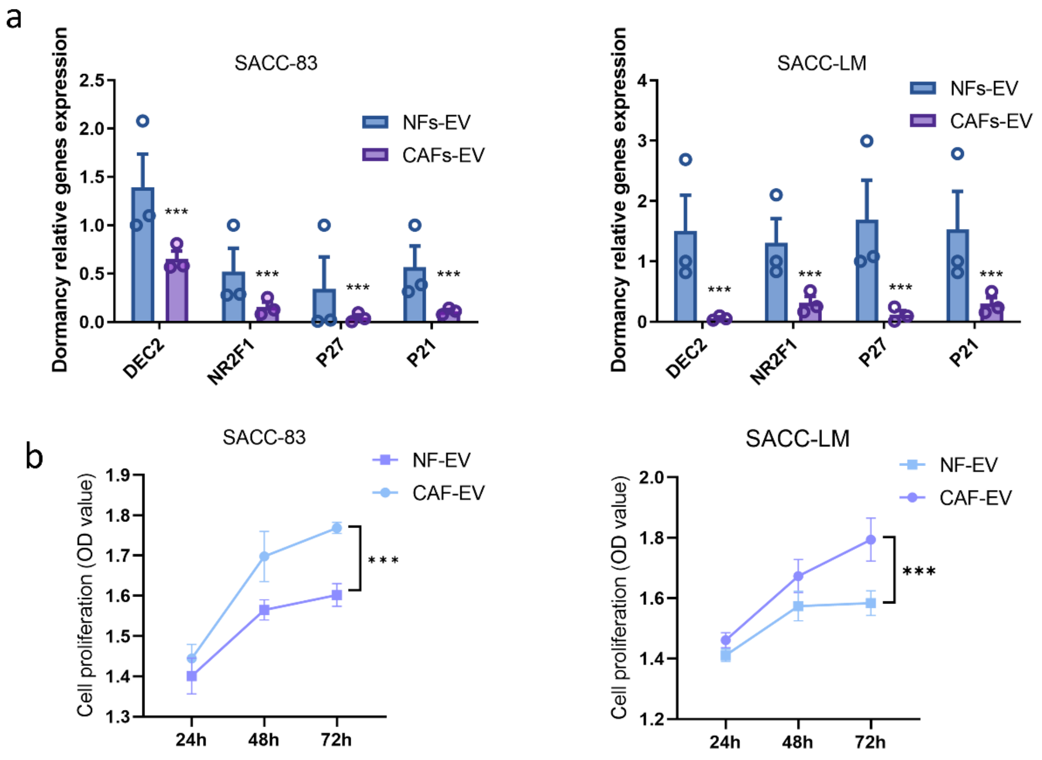


**Supplementary Fig. 5: CAFs-EV contributed to the reactivation of dormancy SACC cells. a** The proliferation assay of dormant SACC cells treated with NFs-EV or CAFs-EV. **b** qRT-PCR measured the relative mRNA level of dormancy in dormant SACC cells treated with NFs-EV or CAFs-EV.

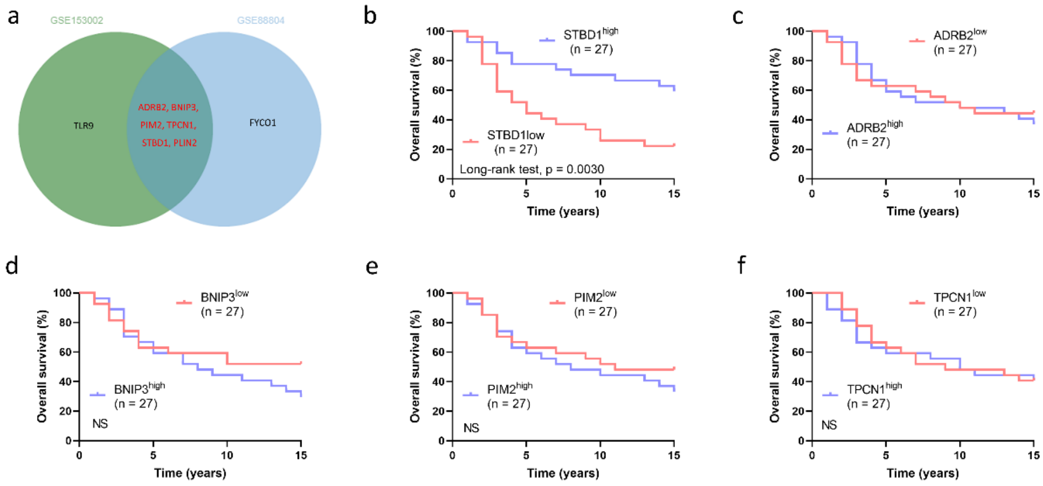


**Supplementary Fig. 6: Correlation between candidate genes and prognosis in patients with SACC. a** Venn diagram of candidate genes in GSE153002 and GSE88804 dataset. **b-f** Kaplan–Meier plots verified the correlation between STBD1,

ADRB2, BNIP3, PIM2 and TPCN1 expression and the overall survival rates in 54 SACC patients.

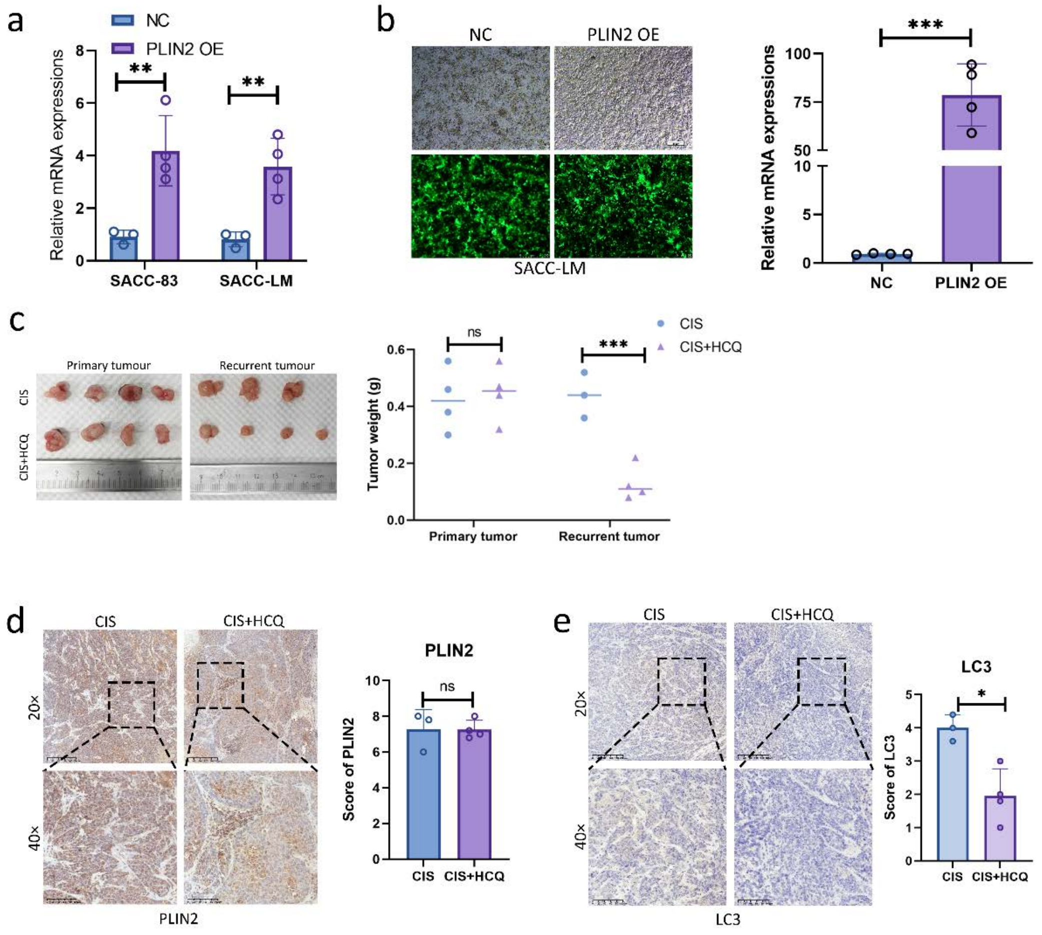


**Supplementary Fig. 7: PLIN2-mediated autophagy induced tumor recurrence. a** Overexpression efficiency test of PLIN2 in dormant SACC-83 and SACC-LM cells. **b** GFP-PLIN2-SACC-LM cells were constructed. **c** Macrograph of primary and recurrent tumors (left), tumor weights were calculated. **d, e** IHC analysis for PLIN2 and LC3 in recurrent tumor tissues.

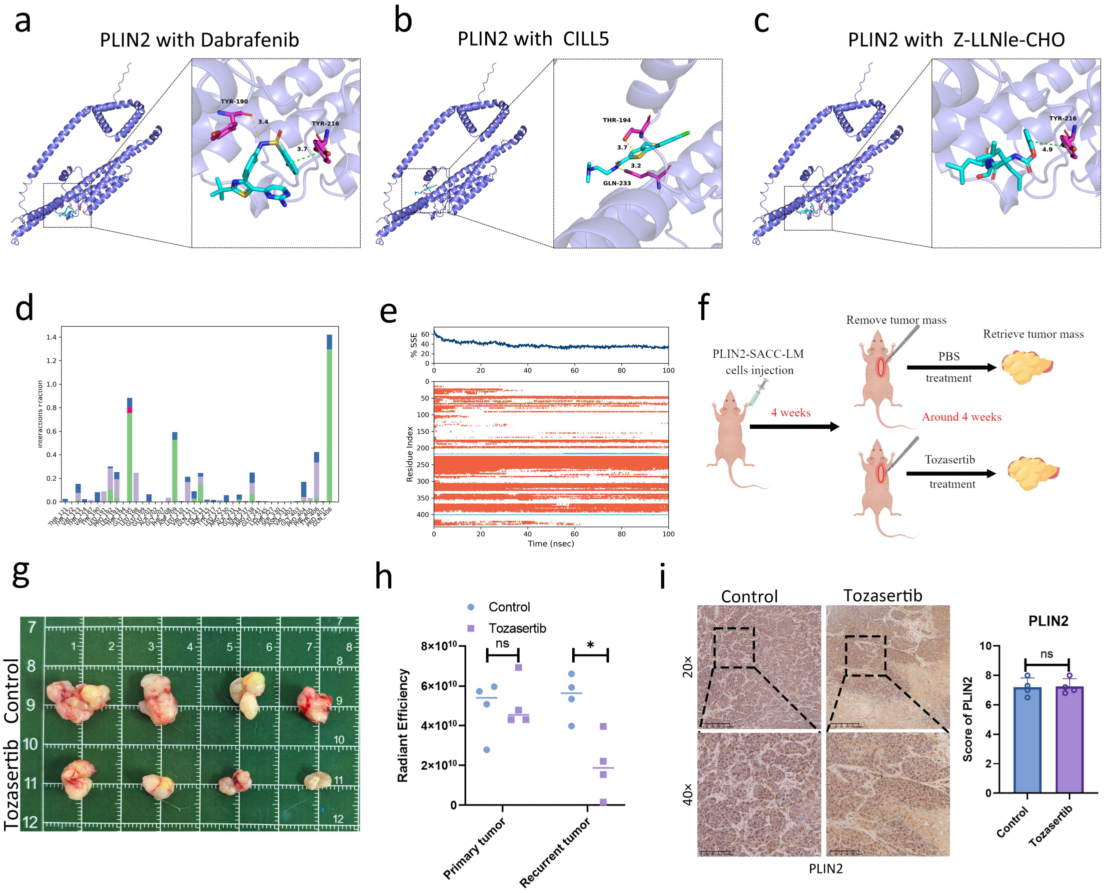


**Supplementary Fig. 8: Tozasertib markedly induced tumor recurrence. a-c**The interactions analysis of PLIN2 and dabrafenib, CIL55 or Z-LLNle-CHO. **d, e** Analysis of the Tozasertib including interactions (d), secondary structural element (e). **f** Schematic diagram of tumor recurrence experiment in mice. **g-h** Macrograph of recurrent tumors (g), the radiant efficiencies of recurrent tumor were calculated (h). **i** IHC analysis for PLIN2 in recurrent tumor tissues.

**Supplementary Tables**

**Supplementary Table 1**

| **Primer Sequence Information** | |
|---|---|
| qPCR: Human GAPDH Forward: | ACAACTTTGGTATCGTGGAAGG |
| qPCR: Human GAPDH Reverse: | GCCATCACGCCACAGTTTC |
| qPCR: Human DEC2 Forward: | CTGATGCTGTTGCTCGGTTA |
| qPCR: Human DEC2 Reverse: | TGCAGACTCTGGGACATCTG |
| qPCR: Human NR2F1 Forward: | GCCTCAAAGCCATCGTGCTG |
| qPCR: Human NR2F1 Reverse: | CCTCACGTACTCCTCCAGTG |
| qPCR: Human P21 Forward: | TAGCAGCGGAACAAGGAG |
| qPCR: Human P21 Reverse: | AAACGGGAACCAGGACAC |
| qPCR: Human P27 Forward: | GGTTAGCGGAGCAATGCGCA |
| qPCR: Human P27 Reverse: | AACCGGCATTTGGGGAACCGTC |
| qPCR: Human Naong Forward: | AGATGCCTCACACGGAGACT |
| qPCR: Human Naong Reverse: | TCTGGAACCAGGTCTTCACC |
| qPCR: Human SOX2 Forward: | CACAACTCGGAGATCAGCAA |
| qPCR: Human SOX2 Reverse: | GTTCATGTGCGCGTAACTGT |
| qPCR: Human Oct4 Forward: | CGAAAGAGAAAGCGAACCAG |
| qPCR: Human Oct4 Reverse: | TGAAGTGAGGGCTCCCATAG |
| qPCR: Human PLIN2 Forward: | TCCACTGTCCACCTGATTGA |
| qPCR: Human PLIN2 Reverse: | TGGCATGTAGTCTGGAGCTG |

**Supplementary Table 2. Clinical parameters of the SACC patients and their association with PLIN2 levels**

| Clinical index | patients (n=48) | PLIN2 | | P vale |
|---|---|---|---|---|
| | | High expression (≥5) | Low expression (<5) | |
| Age | | | | 0.8544 |
| <60 | 30 | 6 | 24 | |
| ≥60 | 18 | 4 | 14 | |
| Gender | | | | 0.8822 |
| male | 25 | 5 | 20 | |
| female | 23 | 5 | 18 | |
| Grade | | | | 0.0102* |
| I-II | 31 | 3 | 28 | |
| III | 17 | 7 | 10 | |
| Recurrence | | | | |
| yes | 10 | 6 | 4 | 0.0006* |
| no | 38 | 4 | 34 | |
